# Hepcidin-guided screen-and-treat interventions for young children with iron-deficiency anaemia in The Gambia: an individually randomised, three-arm, double-blind, controlled, proof-of-concept, non-inferiority trial

**DOI:** 10.1016/S2214-109X(22)00449-1

**Published:** 2022-12-13

**Authors:** Rita Wegmüller, Amat Bah, Lindsay Kendall, Morgan M Goheen, Saikou Sanyang, Ebrima Danso, Ebrima A Sise, Amadou Jallow, Hans Verhoef, Momodou W Jallow, Miriam Wathuo, Andrew E Armitage, Hal Drakesmith, Sant-Rayn Pasricha, James H Cross, Carla Cerami, Andrew M Prentice

**Affiliations:** a Medical Research Council Unit The Gambia at the London School of Hygiene & Tropical Medicine, Banjul, The Gambia; b GroundWork, Fläsch, Switzerland; c National Nutrition Agency, Bakau, The Gambia; d Department of Internal Medicine, Yale University, New Haven, CT, USA; e Wageningen University & Research, Wageningen, Netherlands; f Regeneron Genetics Center, Tarrytown, NY, USA; g One Acre Fund, Kigali, Rwanda; h Medical Research Council Human Immunology Unit, Medical Research Council Weatherall Institute of Molecular Medicine, University of Oxford, John Radcliffe Hospital, Oxford, UK; i Walter and Eliza Hall Institute of Medical Research, Parkville, VIC, Australia

## Abstract

**Background:**

Iron deficiency is the most prevalent nutritional disorder worldwide. Iron supplementation has modest efficacy, causes gastrointestinal side-effects that limit compliance, and has been associated with serious adverse outcomes in children across low-income settings. We aimed to compare two hepcidin-guided screen-and-treat regimens designed to reduce overall iron dosage by targeting its administration to periods when children were safe and ready to receive iron supplementation, with WHO's recommendation of universal iron supplementation.

**Methods:**

We conducted an individually randomised, three-arm, double-blind, controlled, proof-of-concept, non-inferiority trial in 12 rural communities across The Gambia. Eligible participants were children aged 6–23 months with anaemia. Participants were randomly assigned (1:1:1) to either the WHO recommended regimen of one sachet of multiple micronutrient powder (MMP) daily containing 12·0 mg iron as encapsulated ferrous fumarate (control group); to MMP with 12·0 mg per day iron for the next 7 days if plasma hepcidin concentration was less than 5·5 μg/L, or to MMP without iron for the next 7 days if plasma hepcidin concentration was at least 5·5 μg/L (12 mg screen-and-treat group); or to MMP with 6·0 mg per day iron for the next 7 days if plasma hepcidin concentration was less than 5·5 μg/L, or to MMP without iron for the next 7 days if plasma hepcidin concentration was at least 5·5 μg/L (6 mg screen-and-treat group). Randomisation was done by use of a permuted block design (block size of 9), with stratification by haemoglobin and age, using computer-generated numbers. Participants and the research team (except for the data manager) were masked to group allocation. The primary outcome was haemoglobin concentration, with a non-inferiority margin of –5 g/L. A per-protocol analysis, including only children who had consumed at least 90% of the supplements (ie, supplement intake on ≥75 days during the study), was done to assess non-inferiority of the primary outcome at day 84 using a one-sided *t* test adjusted for multiple comparisons. Safety was assessed by use of ex-vivo growth tests of *Plasmodium falciparum* in erythrocytes and three species of sentinel bacteria in plasma samples from participants. This trial is registered with the ISRCTN registry, ISRCTN07210906.

**Findings:**

Between April 23, 2014, and Aug 7, 2015, we prescreened 783 children, of whom 407 were enrolled into the study: 135 were randomly assigned to the control group, 136 to the 12 mg screen-and-treat group, and 136 to the 6 mg screen-and-treat group. 345 (85%) children were included in the per-protocol population: 115 in the control group, 116 in the 12 mg screen-and-treat group, and 114 in the 6 mg screen-and-treat group. Directly observed adherence was high across all groups (control group 94·8%, 12 mg screen-and-treat group 95·3%, and 6 mg screen-and-treat group 95·0%). 82 days of iron supplementation increased mean haemoglobin concentration by 7·7 g/L (95% CI 3·2 to 12·2) in the control group. Both screen-and-treat regimens were significantly less efficacious at improving haemoglobin (–5·6 g/L [98·3% CI –9·9 to –1·3] in the 12 mg screen-and-treat group and –7·8 g/L [98·3% CI –12·2 to –3·5] in the 6 mg screen-and-treat group) and neither regimen met the preset non-inferiority margin of –5 g/L. The 12 mg screen-and-treat regimen reduced iron dosage to 6·1 mg per day and the 6 mg screen-and-treat regimen reduced dosage to 3·0 mg per day. 580 adverse events were observed in 316 participants, of which eight were serious adverse events requiring hospitalisation mainly due to diarrhoeal disease (one [1%] participant in the control group, three [2%] in the 12 mg screen-and-treat group, and four [3%] in the 6 mg screen-and-treat group). The most common causes of non-serious adverse events (n=572) were diarrhoea (145 events [25%]), upper respiratory tract infections (194 [34%]), lower respiratory tract infections (62 [11%]), and skin infections (122 [21%]). No adverse events were deemed to be related to the study interventions.

**Interpretation:**

The hepcidin-guided screen-and-treat strategy to target iron administration succeeded in reducing overall iron dosage, but was considerably less efficacious at increasing haemoglobin and combating iron deficiency and anaemia than was WHO's standard of care, and showed no differences in morbidity or safety outcomes.

**Funding:**

Bill & Melinda Gates Foundation and UK Medical Research Council.

## Introduction

The latest estimates from the Global Burden of Disease 2019 project rank iron deficiency as the most prevalent nutritional disorder worldwide.^1^ In many countries across Africa and Asia, iron deficiency contributes to more years lived with disability than any other health condition. Iron supplements are cheap and easy to deliver, and WHO recommends that young children living in areas where the prevalence of anaemia exceeds 20% should receive 90 sachets of multiple micronutrient powders (MMPs) containing 10·0–12·5 mg iron over 6 months.^2^, ^3^ However, a meta-analysis of randomised trials of iron-containing MMPs showed that the efficacy of such interventions is modest, with a mean increase in haemoglobin of 2·7 g/L (95% CI 2·0–3·5)^4^ and a reduction in anaemia prevalence of 18% (10–24).^4^ Other meta-analyses are broadly similar.^5^

In general, iron supplements are poorly absorbed, with absorption efficiency rarely exceeding 30% and typically being 15% or lower.^6^ Absorption efficiency is a function of the chemical form of the supplemental iron, dietary patterns, and the iron and infection status of the recipient. The influences of iron status and infections are mediated by circulating concentrations of the hormone hepcidin, the master regulator of iron absorption and distribution.^7^ The growth and virulence of many pathogens is stimulated by iron, and hepcidin has evolved to downregulate the absorption and circulation of iron when it detects a threat of infection (signalled by inflammatory cytokines, including interleukin-6).^7^, ^8^ Among children living in unhygienic environments with recurrent infections and chronic inflammation, hepcidin is frequently upregulated,^9^ downregulating iron absorption.^10^ Iron supplements can increase the amount of unabsorbed iron passing to the large intestine, where it can cause dysbiosis,^11^, ^12^, ^13^ gastrointestinal side-effects,^14^ and diarrhoea or constipation.^14^, ^15^ Additionally, supplemental iron might enhance the growth of protozoal and bacterial pathogens—an effect that is assumed to be the cause of the serious adverse outcomes observed in several large-scale iron intervention trials in low-income settings (eg, Tanzania and Pakistan).^15^

If a child has a low circulating concentration of hepcidin, it is likely that they do not have inflammation or an infection that might be exacerbated by oral iron,^7^, ^8^ and that intestinal absorption of oral iron will be efficient.^10^ We reasoned that a low concentration of hepcidin could indicate being safe and ready to receive iron (appendix pp 2–3), and could be used to target iron supplementation more effectively, thus reducing the overall dosage of iron administered and the associated risks. We aimed to compare high-dose and low-dose hepcidin-guided screen-and-treat regimens versus WHO's standard of care (ie, MMPs containing 12·0 mg of iron daily) in young children (aged 6–23 months) living in areas with a high prevalence of anaemia in The Gambia, representing the age group most at risk of iron deficiency.

**Research in context**

**Evidence before this study**

WHO recommends that children living in areas where the prevalence of anaemia exceeds 20% should receive 90 sachets of multiple micronutrient powders containing 10·0–12·5 mg iron over 6 months. Meta-analyses of randomised trials using these and similar protocols conducted under supervised conditions indicate an efficacy of only 5·0 g/L increase in haemoglobin concentration or less. Real-life effectiveness is probably much lower due to a combination of poor implementation and compliance. Compliance is often low because supplemental iron can cause discomfort due to unabsorbed reactive iron in the small gut, dysbiosis in the large gut, or both. Several large trials in low-income settings have also reported an excess of serious adverse outcomes, including diarrhoea and dysentery, respiratory tract infections, malaria, hospitalisations, and deaths. The iron-regulatory hormone, hepcidin, is upregulated by infection and inflammation and downregulates absorption of iron in the duodenum. Low circulating concentrations of hepcidin typically indicate that children do not have infection or inflammation, have iron deficiency, and will absorb iron effectively—ie, that they are safe and ready to receive supplemental iron. We reasoned that a hepcidin-guided screen-and-treat approach might achieve equivalent efficacy to daily iron supplementation but at a lower dose; therefore, it might have an improved profile of side-effects and safety.

**Added value of this study**

We tested two screen-and-treat regimens (12 mg/day and 6 mg/day iron when hepcidin was below a pre-established threshold, and none when above the threshold) versus the WHO standard-of-care approach of 12 mg/day. The screen-and-treat strategy was effective at targeting supplementation and, thus, reducing overall intake of supplemental iron. However, both screen-and-treat approaches were inferior to WHO's recommendation of standard daily iron supplementation at increasing haemoglobin, decreasing prevalence of anaemia, and decreasing prevalence of iron deficiency. There were no differences in morbidity or safety outcomes between the screen-and-treat regimens and the WHO recommended regimen.

**Implications of all the available evidence**

Contrary to conceptual logic, a hepcidin-guided screen-and-treat approach to targeting iron administration was less effective than WHO's recommended practice of universal iron supplementation at 12 mg daily. However, because the standard approach has generally low efficacy, research into improved interventions must continue.

## Methods

### Study design and participants

This individually randomised, three-arm, double-blind, controlled, proof-of-concept, non-inferiority trial was conducted in 12 rural communities divided into four geographical clusters in the Kiang East and Jarra West districts of The Gambia, an area with a high prevalence of anaemia and a low endemicity of malaria. Fieldworkers identified children aged 6–23 months at child welfare clinics at the Soma Health Center and Kaiaf Health Center. After obtaining demographic information and written informed consent from the child's primary caregiver at their homes, children were screened at the nearest health centre. Children were eligible for enrolment if they were deemed to be healthy according to a nurse's physical examination; had Z scores for height, weight, and weight-for-height above –3 SD; had a haemoglobin concentration between 70 and 109 g/L; had a negative rapid diagnostic test for malaria; were resident in the study area; their parents or guardians were willing to comply; did not have any congenital disorders or chronic diseases; were not taking regular medication; and were not participating in another study.

Full details of the study design are in the published trial protocol.^16^ The trial was approved by the Scientific Coordination Committee of the Medical Research Council (MRC) Unit The Gambia and the Joint Gambia Government MRC Ethics Committee (SCC1358, amendments L2014.26 and L2014.49). A data safety monitoring board, trial steering committee, and trial monitor monitored the study and the Clinical Trials Office at the MRC assured that the study was conducted according to good clinical practice.

### Randomisation and masking

At enrolment (day 0), eligible children were randomly allocated (1:1:1) to either the WHO recommended regimen of one sachet of MMP daily containing 12·0 mg iron as encapsulated ferrous fumarate (control group);^2^, ^3^ to MMP with 12·0 mg iron for the next 7 days if plasma hepcidin concentration was less than 5·5 μg/L, or to MMP without iron for the next 7 days if plasma hepcidin concentration was at least 5·5 μg/L (12 mg screen-and-treat group); or to MMP with 6·0 mg iron for the next 7 days if plasma hepcidin concentration was less than 5·5 μg/L, or to MMP without iron for the next 7 days if plasma hepcidin concentration was at least 5·5 μg/L (6 mg screen-and-treat group). Randomisation was done by use of a permuted block design (block size of 9), with stratification by haemoglobin (above and below the median haemoglobin concentration of the respective enrolment day) and age (6–11 months, 12–17 months, and 18–23 months), using computer-generated numbers. Derivation of the hepcidin threshold of 5·5 μg/L to define being ready and safe to receive iron was based on a receiver operating characteristic analysis to define iron deficiency and iron-deficiency anaemia in three large cohorts from The Gambia, Kenya, and Tanzania, and isotopically assessed functional iron absorption in Gambian children as previously described.^17^ Participants and the research team (with the exception of the data manager) were masked to group allocation and supplementation type throughout the fieldwork and data analysis. The field coordinator pre-packed the weekly supplements for each participant using computer-generated lists accounting for each week's preceding hepcidin value in the two screen-and-treat groups.

### Procedures

MMPs were produced under good manufacturing practice by DSM Nutritional Products (Johannesburg, South Africa). The MMPs used in this study contained 15 micronutrients (appendix p 5) and only differed in iron concentration: 0·0 mg, 6·0 mg, and 12·0 mg iron as encapsulated ferrous fumarate per sachet (daily dose). All MMPs were packed in identical sachets to ensure blinding. The content of one sachet was mixed with a small quantity of an orange-flavoured drink (Yandi; Comfort Quality Service, Banjul, The Gambia) by a fieldworker who supervised daily administration at participants’ homes. The first MMP was consumed on day 2 to take into account the hepcidin value from day 0, and continued for 82 days.

Study participants were recruited in cohorts (appendix p 11) and assessed according to the trial design (appendix p 12). At screening (day 0), a nurse examined the child and checked their medical history. A fieldworker measured body length (Seca 417 lengths board, Seca, Hamburg, Germany), weight (Seca 336 baby scale, Seca, Hamburg, Germany), head circumference (Seca CE 0123, Seca, Hamburg, Germany), mid-upper arm circumference (Seca 212, Seca, Hamburg, Germany), and triceps skinfold thickness (Skinfold caliper, Holtain, Crymych, UK). Children with any Z scores (height, weight, or weight-for-height) below –3 SD were excluded before randomisation and referred to the regional health centre for further management. A nurse then took a fingerprick blood sample to assess haemoglobin (HemoCue Hb 301, HemoCue, Ångelholm, Sweden) and to carry out the malaria rapid test (Alere Bioline Malaria Ag Pf, Abbot, Seoul, South Korea). Children with haemoglobin values below 70·0 g/L or above 109·0 g/L, or a positive malaria result were excluded. Children with malaria or haemoglobin values below 70·0 g/L were treated according to national guidelines. A nurse then took a venous blood sample (5 mL divided into EDTA [edetic acid], heparin, and citrate phosphate dextrose adenine monovettes) for further analysis, which was processed and stored at the MRC The Gambia at the London School of Hygiene & Tropical Medicine field station in Keneba, The Gambia. Caregivers were asked to provide a stool sample of the participating child within the following 2 days.

From day 2 to day 84, all children were seen on a daily basis by a fieldworker who supervised administration of the MMP, and recorded and managed any adverse events. Field workers collected data on morbidity twice a week. At day 7 and weekly thereafter (except on days 49 and 84 when venous blood was collected), capillary blood was sampled for the measurement of haemoglobin by the HemoCue analyser, *Plasmodium falciparum* infection by rapid diagnostic test, and plasma hepcidin concentrations (to define subsequent allocation of iron or no iron among children in the screen-and-treat groups). To maintain masking, collection of capillary blood samples and all analyses were also performed in the control group. On days 49 and 84, another venous blood sample was taken and, on days 14 and 84, another stool sample was collected from all participants. Venous blood samples in EDTA collected on days 0, 49, and 84 were used for full blood count (Medonic M Series, Boule Diagnostics, Spanga, Sweden) and for DNA extraction.

Plasma hepcidin concentrations were measured weekly by ELISA with a detection range of 0·049–25·000 μg/L (human hepcidin-25 EIA Kit, Peninsula Laboratories International, San Carlos, CA, USA) on the day of blood collection. Results were available on the following day. The assay was later validated as part of the worldwide harmonisation exercise.^18^ Remaining plasma from venous blood (on days 0, 49, and 84) was stored at –20°C and later used for measuring ferritin, iron, unsaturated iron-binding capacity, transferrin saturation, soluble transferrin receptor (sTfR), C-reactive protein (CRP), and alpha-1-acid glycoprotein 1 (AGP 1) using an automated analyser (Cobas Integra 400 plus, Roche Diagnostics, Rotkreuz, Switzerland) and for ex-vivo bacterial growth assays in heat-inactivated plasma as described previously.^19^ We selected three sentinel bacteria species (*Staphylococcus aureus*, *Salmonella enterica*, and *Escherichia coli*) to represent several iron-acquisition mechanisms and because they frequently cause sepsis in low-income settings. We used freshly washed red blood cells from the citrate phosphate dextrose adenine tubes on days 0, 49, and 84 for ex-vivo *P falciparum* growth assays as described previously.^20^, ^21^ Reticulocyte counts were assessed by fluorescence-activated cell sorting of CD71-positive cells. To provide an independent marker of supplement compliance, washed red blood cells were used to assess riboflavin status at days 0 and 84 by use of the erythrocyte glutathione reductase activation coefficient (EGRAC) index in a pragmatic sample of 143 children with sufficient blood volume.^22^ Stool samples collected on days 0, 14, and 84 were aliquoted and frozen for later analysis of calprotectin by ELISA (fCAL; Bühlmann Laboratories, Schönenbuch, Switzerland) and of stool regenerating gene 1B (REG1B) by ELISA (Virginia Health Systems, Charlottesville, VA, USA).^23^

### Outcomes

The primary endpoint was haemoglobin concentration at day 84 among children in the two screen-and-treat groups versus those in the control group. A secondary aim was to compare haemoglobin concentration among children in the 12 mg screen-and-treat group with those in the 6 mg screen-and-treat group. Secondary endpoints were the proportion of participants with anaemia, iron deficiency, and iron-deficiency anaemia at day 84, the total amount of iron administered over the 12-week study period, morbidity as assessed by twice weekly questionnaires and reporting of adverse events, and safety as assessed by the ex-vivo growth of *P falciparum* and three sentinel bacteria species (*S aureus*, *S enterica*, and *E coli*). Additional secondary outcomes were ferritin adjusted for inflammation, sTfR, transferrin, and transferrin saturation at day 84.

The total dosage of iron was calculated according to the fieldworker record of consumption, multiplied by the iron dose of the respective day, and summed over the whole study period. Episodes of fever, diarrhoea, vomiting, and cough were summed over the 12-week study period for each condition if the caregiver indicated that the child had developed the condition since the previous visit. Adverse events were defined as any untoward or unfavourable medical occurrence, whether considered related to the child's participation in the study or not. Serious adverse events were always investigated by the trial clinician and were defined as adverse events that were life-threatening, resulted in death, required or extended hospitalisation, or resulted in a persistent and significant disability or incapacity.

### Statistical analysis

The sample size calculation was based on a study conducted in children aged 24 months in a neighbouring district, which yielded an SD of 11·5 g/L for haemoglobin concentration.^24^ The findings from this study resulted in a sample size of 131 children per group using a one-sided α of 2·5% and a Bonferroni correction to adjust for multiple testing. Assuming a dropout rate of less than 15%, it was established that a total sample size of 393 children would provide 80% probability that the 95% CI would exclude the non-inferiority margin of –5·0 g/L. To take seasonality into account and to be able to properly manage and monitor the study, we enrolled children into five cohorts, starting on May 26, 2014, Aug 25, 2014, Dec 15, 2014, March 16, 2015, and Aug 10, 2015 (appendix p 11).

We used a per-protocol analysis including only children who had consumed at least 90% of the supplements, corresponding to supplement intake on at least 75 days during the study, to assess non-inferiority of the primary outcome at day 84 using a one-sided *t* test adjusted for multiple comparisons. We also conducted a modified intention-to-treat analysis in all children with a haemoglobin result at day 84 (excluding children withdrawn from the study, when blood sampling was not possible at day 84, or if the samples clotted), which did not take compliance into account. We further simulated a true intention-to-treat analysis in all randomly assigned participants by conducting a linear mixed-effects model analysis, which also took into account baseline values. The effect of the intervention for continuous variables was measured by mixed-effects linear regression, with logarithmic transformation used as appropriate. Log transformation adequately dealt with all variables that were not normally distributed. Linearity and constant variance requirements were met in all analyses. Differences in the proportion of participants with anaemia, iron deficiency, and iron-deficiency anaemia between intervention groups at day 84 were analysed using mixed-effects logistic regression. Data imputation was not performed.

All statistical analysis was done in Stata (version 15.1). This trial is registered with the ISRCTN registry, ISRCTN07210906.

### Role of the funding source

The funders of the study had no role in study design, data collection, data analysis, data interpretation, or writing of the report.

## Results

**Figure 1:**
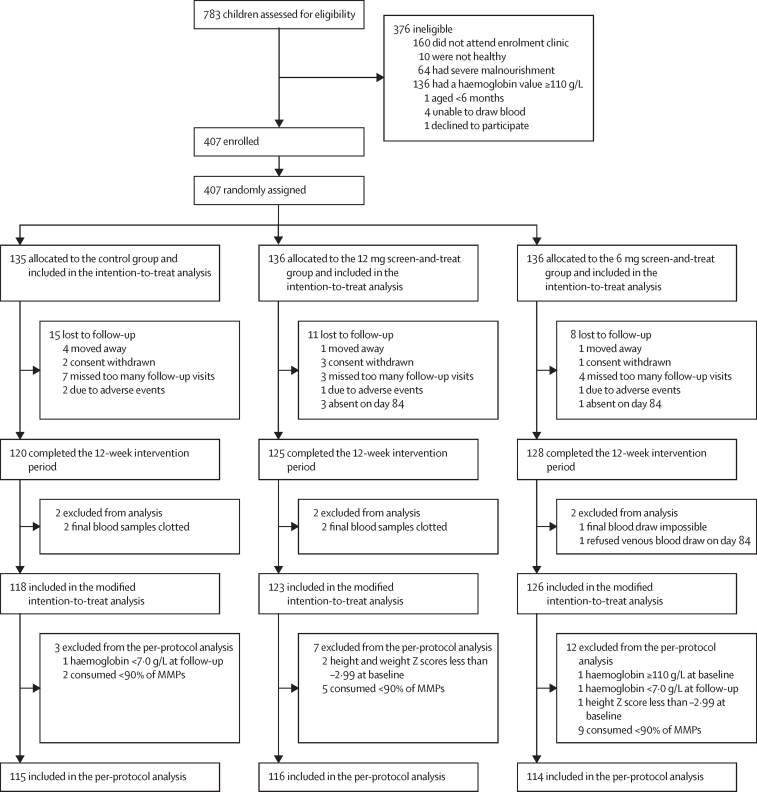
Trial profile MMPs=multiple micronutrient powders.

Between April 26, 2014, and Aug 7, 2015, we prescreened 783 children, of whom 407 were enrolled into five cohorts. Overall, 135 were randomly assigned to the control group, 136 to the 12·0 mg screen-and-treat group, and 136 to the 6·0 mg screen-and-treat group (figure 1; appendix p 11). Mean loss to follow-up was 8·4%, and was slightly higher in the control group. 367 (90%) of 407 children had a haemoglobin result at day 84. The per-protocol population included only children with a compliance of more than 90% MMP consumption, and excluded six children who were mistakenly included in the study because they had either a haemoglobin value slightly above, or a height or weight Z score slightly below, the cutoff value at recruitment, or because they had an untreated haemoglobin value slightly below 70·0 g/L during the study. In total, 345 (85%) enrolled children were included in the per-protocol population: 115 in the control group, 116 in the 12 mg screen-and-treat group, and 114 in the 6 mg screen-and-treat group.

Baseline characteristics were similar between intervention groups, with only minor differences in the proportions of children with underweight or wasting, and in the EGRAC index (table 1; appendix p 6). The overall prevalence of stunting was 14% (n=58 children), underweight was 19% (n=79), and wasting was 13% (n=51; appendix p 6). 358 (88%) of 407 participants had anaemia based on the Medonic automated analyser in the laboratory (407 [100%] with use of the HemoCue field photometer), 209 (57%) of 365 children had iron deficiency, and 192 (53%) of 365 had iron-deficiency anaemia. Almost half (193 [47%]) of enrolled children had a hepcidin concentration below the preset threshold of less than 5·5 μg/L, which was used to determine whether they should receive iron supplementation the following week. Inflammation was common, with 251 (65%) of 388 children with elevated AGP 1 concentrations, but none of the children tested positive for *P falciparum* malaria at baseline. 126 (88%) of 143 children had riboflavin deficiency and gut inflammation was common; 201 (81%) children had a faecal calprotectin concentration above 75 μg/g.

**Table 1:** Baseline characteristics of the intention-to-treat population

			**Control group (n=135)**	**12 mg screen-and-treat group (n=136)**	**6 mg screen-and-treat group (n=136)**
Age, months			15·5 (4·4)	15·4 (4·4)	15·4 (4·5)
Sex					
	Female		71 (53%)	72 (53%)	66 (49%)
	Male		64 (47%)	64 (47%)	70 (51%)
Haematology					
	Haemoglobin concentration, g/L (by Medonic analyser)		97 (90–103)	99 (92–104)	97 (91–104)
	Anaemia (haemoglobin <110 g/L; by Medonic analyser)		123/135 (91%)	119/136 (88%)	116/136 (85%)
	Haematocrit, %		27·7 (25·4–29·1)	27·9 (25·8–29·7)	27·5 (25·4–29·7)
Plasma iron markers					
	Hepcidin, μg/L		4·9 (1·1–24·9)	6·7 (1·4–35·4)	6·8 (1·8–26·5)
	Hepcidin <5·5 μg/L		69 (51%)	60 (44%)	64 (47%)
	Ferritin, μg/L		13·9 (6·4–22·5)	13·9 (4·8–27·0)	11·2 (3·3–25·3)
	Iron deficiency*		69/123 (56%)	65/118 (55%)	75/124 (60%)
	Iron-deficiency anaemia†		64/123 (52%)	57/118 (48%)	71/124 (57%)
	Transferrin, g/L		3·16 (0·62)	3·17 (0·69)	3·17 (0·64)
	Unsaturated iron-binding capacity, μmol/L		64·4 (15·3)	64·4 (16·9)	64·5 (15·7)
	Iron, μmol/L		4·7 (2·8–7·0)	4·9 (2·7–7·4)	5·0 (2·9–7·0)
	Transferrin saturation <6·5%‡		63/117 (54%)	60/113 (53%)	56/111 (50%)
	sTfR, mg/L‡		12·0 (10·0–15·7)	12·8 (10·1–16·7)	12·3 (9·5–16·1)
	CRP, mg/L		2·4 (1·3–4·8)	2·1 (1·2–5·4)	2·2 (1·0–5·0)
	AGP 1, g/L		1·17 (0·92–1·48)	1·18 (0·91–1·53)	1·17 (0·93–1·54)
	CRP >5·0 mg/L		31/128 (24%)	33/128 (26%)	33/132 (25%)
	AGP 1 >1·0 g/L		80/126 (63%)	83/126 (66%)	88/132 (67%)
Vitamin B_2_ status					
	EGRAC		2·08 (0·56)	1·77 (0·37)	1·90 (0·36)
		Data missing	63 (47%)	97 (71%)	104 (76%)
	Low vitamin B_2_ status (EGRAC >1·4)‡		65/72 (90%)	31/39 (79%)	30/32 (94%)
Faecal markers					
	Calprotectin, μg/g (gut inflammation marker)‡		352·8 (128·4–882·3)	242·3 (78·3–560·5)	307·2 (117·0–730·8)
		Data missing	53 (39%)	56 (41%)	50 (37%)
	REG1B, μg/g (gut mucosa integrity marker)‡		51·8 (3·1–200·9)	46·2 (3·1–202·7)	28·8 (6·2–137·5)
		Data missing	75 (56%)	69 (51%)	72 (53%)

Data are mean (SD), n (%), n/N (%), or median (IQR). sTfR=soluble transferrin receptor. CRP=C-reactive protein. AGP 1=alpha-1-acid glycoprotein 1. EGRAC=erythrocyte glutathione reductase activation coefficient. REG1B=regenerating gene 1B.

* Defined as either ferritin <12 μg/L and ferritin index >3·2 if CRP ≤5 mg/L, or ferritin <30 μg/L and ferritin index >2 if CRP >5 mg/L, where ferritin index is sTfR/log ferritin.

† Defined as haemoglobin <110 g/L, ferritin <12 μg/L, and ferritin index >3·2 if CRP ≤5 mg/L; or haemoglobin <110 g/L, ferritin <30 μg/L, and ferritin index >2 if CRP >5 mg/L.

‡ Adjustments and cutoffs are provided in the appendix (p 4).

**Figure 2:**
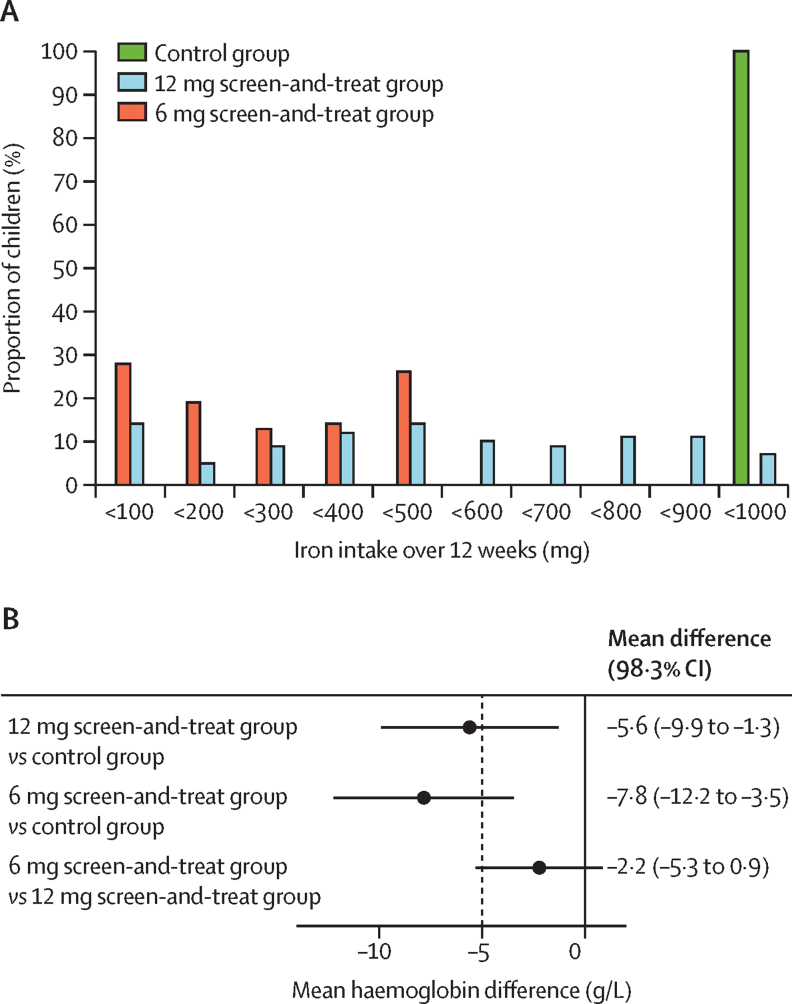
Iron intake and non-inferiority analysis (A) Total iron intake by intervention group. (B) Non-inferiority analysis. Dotted line indicates the predefined margin of –5 g/L; error bars indicate 98·3% CI.

The directly observed compliance with daily iron supplementation was high and similar across all intervention groups: 94·8% in the control group, 95·3% in the 12 mg screen-and-treat group, and 95·0% in the 6 mg screen-and-treat group. In the per-protocol population, the iron dose consumed over the 12-week study period ranged from 900 mg to 996 mg in the control group (mean 984 mg), from 0 mg (eight participants) to 996 mg (one participant) in the 12 mg screen-and-treat group (500 mg), and from 0 mg (seven participants) to 498 mg (three participants) in the 6 mg screen-and-treat group (246 mg; appendix p 7; figure 2A). Compared with the daily intake of 12·0 mg iron in the control group, the 12 mg screen-and-treat regimen reduced iron dosage to 6·1 mg/day and the 6 mg screen-and-treat regimen reduced dosage to 3·0 mg per day. The high compliance rates were cross-validated by the fact that the mean prevalence of riboflavin deficiency across all intervention groups decreased from 88% at baseline to 31% at 12 weeks (data not shown).

In the control group, 82 days of supervised iron supplementation increased mean haemoglobin concentration by 7·7 g/L (95% CI 3·2 to 12·2) in the per-protocol population (table 2). For both screen-and-treat groups, mean haemoglobin concentrations at day 84 showed that both regimens were significantly less efficacious than standard of care: –5·6 g/L (98·3% CI –9·9 to –1·3) in the 12 mg screen-and-treat group and –7·8 g/L (–12·2 to –3·5) in the 6 mg screen-and-treat group (figure 2B), and neither regimen met the pre-set non-inferiority margin of –5 g/L. The modified intention-to-treat analysis, including all participants with a haemoglobin value at day 84, showed similar results: –5·6 g/L (–9·8 to –1·3) in the 12 mg screen-and-treat group and –7·7 g/L (–12·0 to –3·4) in the 6 mg screen-and-treat group. The intention-to-treat analysis, including all randomly assigned participants, also resulted in similar differences: –5·4 g/L (–9·7 to –1·1) in the 12 mg screen-and-treat group and –7·6 g/L (–11·9 to –3·3) in the 6 mg screen-and-treat group. When comparing the two screen-and-treat regimens, we found no evidence that the 6 mg regimen was inferior to the 12 mg regimen (per-protocol difference –2·2 g/L [–5·3 to 0·9]; figure 2B), but we did not show non-inferiority for the 6 mg regimen.

**Table 2:** Primary outcomes, secondary outcomes, and continuous variables at day 84 in the per-protocol analysis

	**Participants with available data**	**Estimate (95% CI)**	**Effect (95% CI)**
**Haemoglobin**			
Control group	115/135 (85%)	104·4 g/L (100·7 to 108·1*)	..
12 mg screen-and-treat group	116/136 (85%)	98·8 g/L (96·7 to 100·9*)	−5·6 g/L (−9·9 to −1·3*)
6 mg screen-and-treat group	114/136 (84%)	96·6 g/L (94·4 to 98·8*)	−7·8 g/L (−12·2 to −3·5*)
**Hepcidin**			
Control group	115/135 (85%)	9·4 μg/L (6·5 to 13·6)	..
12 mg screen-and-treat group	114/136 (84%)	8·1 μg/L (5·6 to 11·8)	−13·8% (−48·8 to 45·2)
6 mg screen-and-treat group	112/136 (82%)	5·8 μg/L (4·0 to 8·4)	−39·0% (−63·8 to 2·9)
**Ferritin**			
Control group	104/135 (77%)	12·1 μg/L (8·8 to 16·7)	..
12 mg screen-and-treat group	109/136 (80%)	13·9 μg/L (10·1 to 19·1)	14·7% (−26·8 to 79·6)
6 mg screen-and-treat group	108/136 (79%)	7·2 μg/L (5·2 to 10·0)	−40·5% (−62·2 to −6·2)
**Inflammation-adjusted ferritin**			
Control group	78/135 (58%)	8·6 μg/L (5·9 to 12·6)	..
12 mg screen-and-treat group	75/136 (55%)	11·3 μg/L (7·7 to 16·7)	31·2% (−23·6 to 125·3)
6 mg screen-and-treat group	81/136 (60%)	6·3 μg/L (4·4 to 9·1)	−26·9% (−56·7 to 23·3)
**sTfR**			
Control group	114/135 (84%)	10·2 mg/L (9·6 to 10·8)	..
12 mg screen-and-treat group	113/136 (83%)	11·5 mg/L (11·0 to 12·1)	13·1% (4·4 to 22·4)
6 mg screen-and-treat group	110/136 (81%)	12·7 mg/L (11·9 to 13·5)	24·2% (13·8 to 35·5)
**Ferritin index**			
Control group	113/135 (84%)	4·8 (4·0 to 5·9)	..
12 mg screen-and-treat group	112/136 (82%)	4·9 (4·2 to 5·7)	1·2% (−21·4 to 30·2)
6 mg screen-and-treat group	107/136 (79%)	7·7 (6·1 to 9·8)	60·3% (17·5 to 118·8)
**Transferrin**			
Control group	114/135 (84%)	2·88 g/L (2·78 to 2·98)	..
12 mg screen-and-treat group	113/136 (83%)	3·08 g/L (2·98 to 3·18)	0·20 g/L (0·06 to 0·34)
6 mg screen-and-treat group	110/136 (81%)	3·16 g/L (3·04 to 3·27)	0·28 g/L (0·13 to 0·43)
**Unsaturated iron-binding capacity**			
Control group	113/135 (84%)	55·5 μmol/L (52·7 to 58·3)	..
12 mg screen-and-treat group	113/136 (83%)	63·8 μmol/L (61·4 to 66·2)	8·3 μmol/L (4·7 to 12·0)
6 mg screen-and-treat group	108/136 (79%)	66·7 μmol/L (64·1 to 69·2)	11·1 μmol/L (7·4 to 14·9)
**Plasma iron**			
Control group	114/135 (84%)	13·8 μmol/L (12·6 to 15·1)	..
12 mg screen-and-treat group	113/136 (83%)	10·3 μmol/L (9·5 to 11·3)	−25·0% (−33·9 to −15·0)
6 mg screen-and-treat group	109/136 (80%)	9·7 μmol/L (9·0 to 10·5)	−29·1% (−37·1 to −20·1)
**Transferrin saturation**			
Control group	114/135 (84%)	19·3 mg/L (17·5 to 21·3)	..
12 mg screen-and-treat group	113/136 (83%)	13·5 mg/L (12·3 to 14·7)	−0·36 mg/L (−0·50 to −0·23)
6 mg screen-and-treat group	109/136 (80%)	12·5 mg/L (11·5 to 13·6)	−0·43 mg/L (−0·56 to −0·30)
**CRP**			
Control group	114/135 (84%)	1·63 g/L (1·24 to 2·15)	..
12 mg screen-and-treat group	113/136 (83%)	1·63 g/L (1·20 to 2·23)	29·2% (−33·8 to 52·0)
6 mg screen-and-treat group	110/136 (81%)	1·28 g/L (0·97 to 1·69)	−21·4% (−46·9 to 16·4)
**AGP 1**			
Control group	114/135 (84%)	1·16 mg/L (1·09 to 1·23)	..
12 mg screen-and-treat group	113/136 (83%)	1·16 mg/L (1·09 to 1·23)	0·05% (−8·30 to 9·20)
6 mg screen-and-treat group	110/136 (81%)	1·10 mg/L (1·04 to 1·17)	−4·50% (−12·20 to 3·90)
**EGRAC**			
Control group	68/135 (50%)	1·32 (1·28 to 1·36)	..
12 mg screen-and-treat group	36/136 (26%)	1·33 (1·28 to 1·38)	0·5% (−4·2 to 5·5)
6 mg screen-and-treat group	29/136 (21%)	1·33 (1·28 to 1·39)	1·1% (−3·7 to 6·2)
**Calprotectin**			
Control group	69/135 (51%)	217·0 μg/g (160·6 to 293·1)	..
12 mg screen-and-treat group	75/136 (55%)	190·5 μg/g (142·6 to 254·4)	−12·2% (−42·2 to 33·3)
6 mg screen-and-treat group	73/136 (54%)	211·1 μg/g (157·3 to 283·1)	−2·7% (−36·1 to 48·1)
**REG1B**			
Control group	53/135 (39%)	26·1 μg/g (14·0 to 48·4)	..
12 mg screen-and-treat group	63/136 (46%)	58·4 μg/g (33·3 to 102·3)	124·0% (−2·8 to 416·0)
6 mg screen-and-treat group	58/136 (43%)	52·6 μg/g (29·1 to 94·9)	101·8% (−14·2 to 374·2)

Data are n/N (%) unless otherwise indicated. Results are from a mixed-effects linear regression, including baseline values with study group as fixed-effect and participant as random-effect factor; log-transformed data for sTfR, ferritin index (sTfR/log ferritin), plasma iron, transferrin saturation, CRP, AGP 1, EGRAC; Tobit regression with log-transformed data for hepcidin, ferritin, ferritin adjusted for inflammation (CRP <5·0 mg/L), calprotectin, and REG1B. All log-transformed data were exponentiated for presentation of estimates as geometric means in the table. Effect sizes are unadjusted and are presented as absolute effects for non-transformed variables and as relative effects (percentage change in geometric mean relative to the control group) for log-transformed variables. EGRAC was measured in a random subsample. sTfR=soluble transferrin receptor. CRP=C-reactive protein. AGP 1=alpha-1-acid glycoprotein 1. EGRAC=erythrocyte glutathione reductase activation coefficient. REG1B= regenerating gene 1B.

* 98·3% CI for primary outcome considering comparison of all three intervention groups, adjusting for multiple testing using a one-sided test. 95% CI for the secondary outcomes, which have not been corrected for multiple testing because they represent alternate measures of related outcomes.

In combined analyses across all intervention groups, the total amount of iron administered significantly (but weakly) predicted changes between baseline and day 84 in haemoglobin (R^2^ 0·05; p=0·0010; coefficient 0·001 g/L per mg iron), ferritin (R^2^ 0·05; p=0·0012; coefficient 0·02 μg/L per mg iron), and transferrin saturation (R^2^ 0·12; p<0·0001; coefficient 0·01% per mg iron). Adjusting for the observed weight gained and assuming a blood volume of 65 mL/kg, the gain of 7·7 g/L haemoglobin would contain an estimated total of 15·3 mg of iron, which represents 1·6% of the mean total of 984·0 mg received by children in the control group. Gains in the two screen-and-treat groups were trivial.

The prevalence of anaemia remained high in all study groups after the 12-week supplementation period (table 3). Although the prevalence decreased by 14 percentage points in the control group from 91% to 77%, it stayed at a similar level in the 12 mg screen-and-treat group (89% to 86%) and increased by 8 percentage points in the 6 mg screen-and-treat group (85% to 93%). Similarly, the prevalence of iron deficiency decreased in the control group from 55% to 35%, but remained at a similar level for the two screen-and-treat groups (table 3). The prevalence of iron-deficiency anaemia did not change within the two screen-and-treat groups, but dropped from 50% to 31% in the control group (table 2).

**Table 3:** Secondary outcomes as categorical variables in the per-protocol analysis

	**Participants with available data**	**Effect (95% CI)**
**Anaemia at day 84 (haemoglobin <110 g/L; by Medonic analyser)**		
Control group	88/115 (77%)	..
12 mg screen-and-treat group	100/116 (86%)	9·7 (−0·3 to 19·7)
6 mg screen-and-treat group	106/114 (93%)	16·5 (7·4 to 25·5)
Iron deficiency at day 84*		
Control group	39/113 (35%)	..
12 mg screen-and-treat group	62/113 (55%)	20·4 (7·7 to 33·0)
6 mg screen-and-treat group	61/108 (56%)	22·0 (9·2 to 43·3)
**Iron-deficiency anaemia at day 84**†		
Control group	35/113 (31%)	..
12 mg screen-and-treat group	52/113 (46%)	15·0 (2·5 to 27·6)
6 mg screen-and-treat group	57/108 (53%)	21·8 (9·1 to 34·5)

Data are n/N (%) unless otherwise indicated. Results are from a mixed-effects logistic regression with study group as fixed-effect and participant as random-effect factor. CRP=C-reactive protein. sTfR=soluble transferrin receptor.

* Defined as either ferritin <12 μg/L and ferritin index >3·2 if CRP ≤5 mg/L, or ferritin <30 μg/L and ferritin index >2 if CRP >5 mg/L, where ferritin index is sTfR/log ferritin.

† Defined as haemoglobin <110 g/L, ferritin <12 μg/L, and ferritin index >3·2 if CRP ≤5 mg/L; or haemoglobin <110 g/L, ferritin <30 μg/L, and ferritin index >2 if CRP >5 mg/L.

Serum ferritin (adjusted for inflammation), hepcidin, CRP, AGP1, EGRAC, faecal calprotectin, and REG1B concentrations in the two screen-and-treat groups did not differ from the control group at the end of the study period (table 2). All other markers of iron status (ie, sTfR, transferrin, plasma iron, unsaturated iron-binding capacity, and transferrin saturation) indicated significantly poorer responses to supplementation in the two screen-and-treat groups than in the control group. Additional haematological parameters at day 84 showed mixed responses, with red blood cell distribution width, mean corpuscular haemoglobin concentration, and white blood cell count indicating no differences between groups and mean corpuscular volume and mean corpuscular haemoglobin showing better values than in the 6 mg screen-and-treat control group (table 2; appendix pp 8–9). The two screen-and-treat groups only differed from each other in the ferritin index and in ferritin concentration unadjusted for inflammation, for which the 12 mg regimen was superior to the 6 mg regimen.

Monitoring morbidity twice a week, asking specifically about the presence of fever, diarrhoea, vomiting, cough, or any other symptom, did not show any differences between intervention groups (appendix p 10). During the study, only three (1%) participants had a positive result on the rapid test for *P falciparum*, all of whom were in the control group.

Over the whole study period, we observed 580 adverse events in 316 participants, of which eight were serious adverse events requiring hospitalisation mainly due to diarrhoeal disease (one [1%] participant in the control group, three [2%] in the 12 mg screen-and-treat group, and four [3%] in the 6 mg screen-and-treat group; appendix p 10). No adverse events were deemed to be related to study interventions and all adverse events were resolved. Of the 572 non-serious adverse events in 314 participants, the most common causes were diarrhoea (145 [25%] of 572 events), upper respiratory tract infections (194 [34%] events), lower respiratory tract infections (62 [11%] events), and skin infections (122 [21%] events). The distribution of non-serious adverse events between intervention groups was similar: 102 (76%) of 135 children in the control group had at least one adverse event (incidence rate 6·3 cases per person-year [95% CI 5·4–7·3]), 111 (82%) of 136 children in the 12 mg screen-and-treat group (6·3 cases per person-year [5·5–7·3]), and 103 (76%) of 136 children in the 6 mg screen-and-treat group (6·6 cases per person-year [5·7–7·5]). Mixed-effects logistic regression models showed that the odds of having a diarrhoeal disease were higher among participants in the 6 mg screen-and-treat group (1·69 [95% CI 1·01–2·83]) than in the control group, with no differences between the two screen-and-treat groups and the control group for any other category of adverse event (appendix p 10). The diarrhoea result disappeared after adjustment for multiple testing.

**Figure 3:**
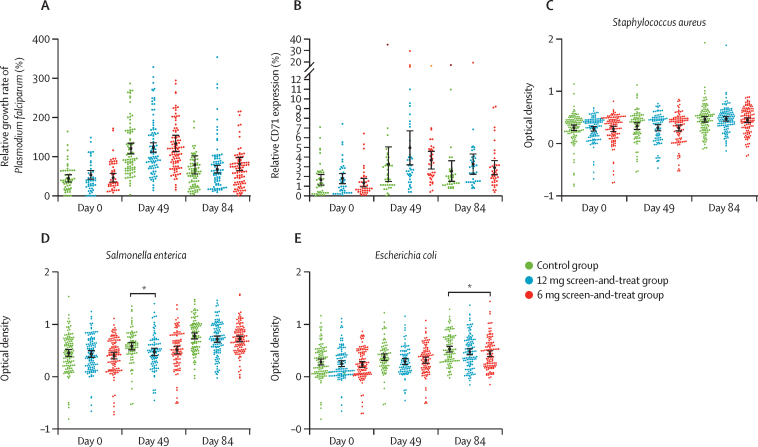
Ex-vivo assays of malaria growth in erythrocytes, reticulocyte count, and sentinel bacteria growth in plasma samples at days 0, 49, and 84 Growth rates of *Plasmodium falciparum* strain FCR3_FMG in erythrocytes (A), reticulocyte count (ie, CD71 expression) compared with non-anaemic controls (B), and growth rates of *Staphylococcus aureus* (C), *Salmonella enterica* (D), and *Escherichia coli* (E) in plasma samples from participants in the control group, the 12 mg screen-and-treat group, and the 6 mg screen-and-treat group. Black lines show mean values and error bars represent 95% CI. *p<0·05.

Compared with blood samples from control participants without anaemia used in the standardised assays, the growth of malaria parasites in fresh red blood cells from participants with anaemia was suppressed at day 0. At day 49, growth was stimulated in all groups and was significantly higher than at baseline (p=0·0012), before a subsequent reduction until day 84, although this still remained higher than at baseline (p=0·0054; figure 3A). No differences were found in malaria growth between intervention groups. CD71 expression (a marker for reticulocytes) paralleled the growth pattern of malaria (figure 3B).

The ex-vivo growth rate of *S aureus* did not differ between days 0 and 49, but was significantly faster at day 84 (p=0·0012; figure 3C). For *S enterica* and *E coli,* growth rate was also faster at day 84 than at both baseline and at day 49 (p=0·0014), and at day 49 compared with baseline (*S enterica* p=0·0033 [figure 3D] and *E coli* p=0·021 [figure 3E]) in serum samples from both the control group and the 6 mg screen-and-treat group. However, samples from the 12 mg screen-and-treat group did not show a difference in growth over the study period. For *S aureus,* no differences were noted between all three intervention groups (figure 3C). For *S enterica*, a faster growth rate was seen in serum samples from children in the control group than in samples from those in the 12 mg screen-and-treat group at day 49 (figure 3D). Similarly, *E coli* grew faster in serum samples from children in the control group than in samples from those in the 6 mg screen-and-treat group on day 84 (figure 3E).

## Discussion

Improved diets with better access to iron-rich foods, as well as environmental improvements aimed at reducing infections and inflammation in early childhood, remain the cornerstone of strategies to reduce the high prevalence of anaemia in many low-income settings. In regions where anaemia prevalence still exceeds 40%, WHO recommends provision of daily multiple micronutrient sachets containing 10·0–12·5 mg elemental iron with a target of at least 90 doses per 6 months in children aged 6–23 months and 2–12 years; however, there are concerns that iron supplementation could increase the risk of infection.^8^, ^15^, ^25^ In this trial, we tested whether a hepcidin-guided screen-and-treat approach could reduce the iron dosage given, while maintaining a non-inferior efficacy. Targeting iron supplements to periods when low hepcidin concentrations indicated that children were ready and safe to receive iron successfully reduced the overall load of supplemental iron by 50% in the 12 mg/day screen-and-treat group and by 75% in the 6 mg/day screen-and-treat group, but was less efficacious than untargeted supplementation for the primary endpoint (haemoglobin concentration) and many secondary endpoints (including prevalence of anaemia and iron deficiency). Neither screen-and-treat approach had discernible advantages in terms of adverse effects or the proxy measures of infection risk (eg, growth rates of malaria and sentinel bacteria) over the standard of care. These results mirror our previous findings in pregnant women with anaemia.^26^

Despite the fact that its efficacy is often low and that side-effects are common, iron is probably the most commonly used preventive and therapeutic agent worldwide. In low-income settings, health systems are forced to use low-cost iron salts. In children, these salts are generally poorly absorbed due to both villous atrophy (because the iron-absorbing divalent metal transporter is active at the tip of the villus^27^) and hepcidin-mediated downregulation of iron absorption secondary to infection and chronic inflammation.^7^, ^8^ The iron not absorbed in the duodenum passes to the large gut, where it can cause a disturbance in the normal gut flora (ie, dysbiosis), which leads to many of the side-effects.^11^, ^12^, ^13^ Additionally, several large randomised trials have reported an excess of serious adverse events following iron supplementation (eg, malaria, severe diarrhoea and dysentery, respiratory tract infections, hospitalisations, and death^28^, ^29^, ^30^, ^31^). Probable mechanisms of these effects have been identified using ex-vivo assays of pathogen growth rates in red blood cells and in plasma samples from children and adults who have received iron supplementation.^21^, ^22^, ^23^

In the current study, we reasoned that we could achieve a similar efficacy at a lower daily dose of iron (hence reducing spillover to the large gut) using two hepcidin-guided screen-and-treat regimens to target periods when iron absorption would be most efficient. We used a previously derived hepcidin threshold to identify periods when children would be ready and safe to receive iron.^17^ We tested these screen-and-treat regimens using the same dose of iron as the WHO standard of care (12 mg) and a half dose (6 mg) in the form of ferrous fumarate. On average, children spent around 50% of the time with hepcidin above the threshold for withholding iron; therefore, we could successfully halve the amount of iron administered in the 12 mg screen-and-treat group and halve it again in the 6 mg screen-and-treat group.

We set a non-inferiority margin of –5 g/L as the clinically acceptable amount by which a lower efficacy would be tolerated if the regimen had counterbalancing advantages of fewer side-effects and a better safety profile. By use of the primary endpoint of haemoglobin value at day 84, the screen-and-treat regimens failed the formal non-inferiority test and were demonstrably worse than the WHO recommendation of daily iron supplementation. The screen-and-treat regimens were also less effective than daily supplementation at resolving anaemia, iron deficiency, and iron-deficiency anaemia. Several key measures of iron status (sTfR, transferrin, plasma iron, unsaturated iron-binding capacity, and transferrin saturation) were also indicative of significantly lower iron status in the two screen-and-treat groups than in the control group. Our analogous trial in pregnant women with anaemia passed the non-inferiority test, but also showed no advantages over the standard WHO recommendation of universal daily iron.^26^

Despite the daily supplements being administered by fieldworkers and a high compliance rate of 95%, the WHO standard of care still showed modest efficacy with an increase in haemoglobin of 7·7 g/L (from 97·0 g/L to 104·0 g/L) and a reduction in anaemia of 14 percentage points (from 91% to 77%) over 12 weeks. This finding is likely to be due to the relatively short intervention period of 12 weeks and the probable wide range of causes of anaemia (including haemoglobinopathies), which are only partly directly attributable to iron deficiency. Of note, there was a greater reduction in iron deficiency in the standard of care group and the haemoglobin response probably showed a lag. We estimate that only 1·6% of the total iron administered in the control group was accrued as haemoglobin. These results are typical of similar trials elsewhere in low-income and middle-income countries.^4^, ^5^ For example, a trial in Bangladesh showed an improvement in haemoglobin of 4·7 g/L among participants with anaemia following 3 months of treatment with MMPs, compared with an improvement of 8·9 g/L with placebo.^32^ These findings align with estimates of the high costs per disability-adjusted life-year averted with iron-containing MMPs.^25^ The screen-and-treat regimens in the current study showed close to no efficacy in reducing the prevalence of anaemia with 12 mg iron daily—from 88% at baseline to 86% at day 84—and an actual increase in prevalence with 6 mg daily—from 85% to 93%. These results reflect a failure to overcome the usual age-related decrease in haemoglobin concentration in this population.

We calculated that only 1·6% of the total 984 mg iron administered to children in the control group was accrued in haemoglobin. In a substudy of 15 children aged 17 months from the control group of the current study, we used a novel ^57^Fe-based method to assess iron absorption over the 84 days of iron administration.^33^ Mean absorption was just 1·0 mg/day (8·3% of the supplemental iron) and, due to elevated losses during supplementation, net gain was just 0·30 mg/day. The estimated iron accrued in haemoglobin was 19·2% of the estimated amount absorbed and 64·0% of the net gain. Therefore, the low efficacy of supplemental iron is almost entirely attributable to poor absorption, and our strategy of targeting iron supplements to periods of increased absorption (indicated by low concentrations of circulating hepcidin) did not overcome this limitation.

Our results indicate that the 6 mg screen-and-treat regimen would not be acceptable under any circumstances. The poorer performance of the 12 mg screen-and-treat regimen might be acceptable if it had a superior profile of side-effects and safety than the WHO standard of care, which would permit long-term supplementation and a gradual resolution of anaemia. However, this situation was not the case; there was no evidence of differences in childhood illnesses reported by the caregiver, adverse or serious adverse events, or in the proxy safety assays of malaria parasite growth in erythrocytes nor in sentinel bacteria growth in plasma between the screen-and-treat regimens and the WHO recommended regimen.

This study has several strengths. First, the trial was conducted in children with anaemia living in the kind of rural, low-income environment representative of many areas where innovative approaches to anaemia prevention and treatment are needed. Second, the study adhered to strict clinical trial standards, the supplements were administered directly, and adherence to supplementation was high. Third, there was a large number of reported illnesses and adverse events, allowing for the detection of group differences if they existed. Fourth, the hepcidin-directed screening protocol achieved the desired reduction in iron dosing (halved in the 12 mg screen-and-treat group and halved again in the 6 mg screen-and-treat group). Finally, the study used innovative ex-vivo safety assays developed and validated in our laboratory.^19^, ^20^, ^21^ Their validity was underlined in this study by the fact that the growth rates of *P falciparum* transiently increased after iron supplementation (driven by enhanced growth in reticulocytes) and diminished again after the reticulocytosis had waned, exactly as observed in our previous study in pregnant women with anaemia.^26^ Likewise, the growth rates of three sentinel species of bacteria progressively increased as supplementation progressed and correlated with plasma concentrations of iron, again as observed previously.^26^

The study was arguably weakened by being conducted in an area of very low malaria prevalence, which precluded testing of altered susceptibility to clinical malaria. Additionally, the weak association between the amount of iron administered and the haematological response could be viewed as a limitation to the study, but is a consequence of the small amount of iron accrued.

There are several reasons that could explain why the notion of targeting iron administration to children who were most likely to absorb more iron, and least likely to be adversely affected, did not work in practice. One explanation might be that the weekly measures of hepcidin concentration were not frequent enough to capture the daily (or even within-day) dynamics of enterocyte iron flux mediated by hepcidin. Nevertheless, studies of diurnal and between-day hepcidin responses to iron administration do not support this explanation.^34^ In a previous analysis of the same hepcidin data from the current study, we found that each child's hepcidin response tended to be constant over time, suggesting a role for other mediators.^9^ Furthermore, even if a cheap point-of-care hepcidin assay was available for use in a scale-up of the putative screen-and-treat approach, it would neither be affordable nor practical to assess hepcidin at shorter intervals. An alternative explanation is simply that high, unphysiological doses of iron salts overwhelm the evolved mechanism of hepcidin-mediated regulation of iron absorption at the enterocyte.

In conclusion, despite the conceptual elegance of a hepcidin-guided screen-and-treat approach, this approach is inferior to WHO's recommendation of universal iron distribution in practice. Of note, even in this context of high adherence to the regimen, the current WHO protocol still only had relatively low efficacy. The search for more effective strategies to combat anaemia in children across low-income settings with high exposure to pathogens must continue, and will probably require a combination of iron compounds with better absorption and fewer side-effects, together with parallel measures to reduce environmental contamination, infection, and persistent low-grade inflammation.

## Data sharing

The study protocol is included in the appendix (pp 13–54). De-identified individual participant data that underlie the results reported in this Article and other material (eg, study protocol) will be available to researchers who provide a methodologically sound proposal, immediately following publication. All data requests should be submitted to the corresponding author for consideration.

## Declaration of interests

We declare no competing interests.
