# Supplementary material for: Hepcidin-guided screen-and-treat interventions for young children with iron-deficiency anaemia in The Gambia: an individually randomised, three-arm, double-blind, controlled, proof-of-concept, non-inferiority trial
Source: Lancet Glob Health. 2022 Dec 13;11(1):e105–16. doi: 10.1016/S2214-109X(22)00449-1 (PMC9764454; doi:10.1016/S2214-109X(22)00449-1)
Supplement: Supplementary appendix [file mmc1.pdf]

THE LANCET
Global Health

Supplementary appendix

This appendix formed part of the original submission and has been peer reviewed. We post it as supplied by the authors.

Supplement to: Wegmüller R, Bah A, Kendall L, et al. Hepcidin-guided screen-and-treat interventions for young children with iron-deficiency anaemia in The Gambia: an individually randomised, three-arm, double-blind, controlled, proof-of-concept, non-inferiority trial. *Lancet Glob Health* 2023; **11:** e105–16.

Hepcidin-guided screen-and-treat interventions for iron-deficiency anaemia in young children: a proof-of-concept, randomised controlled trial in The Gambia

Rita Wegmüller PhD, Amat Bah PhD, Lindsay Kendall MSc, Morgan M Goheen MD/PhD, Saikou Sanyang, Ebrima Danso MSc, Ebrima A Sise MSc, Amadou Jallow MSc, Hans Verhoef PhD, Momodou W Jallow PhD, Miriam Wathuo MSc, Andrew E Armitage DPhil, Prof Hal Drakesmit PhD, Prof Sant-Rayn Pasricha MD/PhD, James H Cross PhD, Carla Cerami MD/PhD, Prof Andrew M Prentice PhD

Correspondence to: Prof Andrew Prentice, MRC Unit The Gambia @ LSHTM, Atlantic Boulevard, Fajara, Banjul, The Gambia Email: Andrew.prentice@lshtm.ac.uk or aprentice@mrc.gm Tel: +220-7236903

SUPPLEMENTARY MATERIALS

Rationale for the hepcidin-guided screen-and-treat approach to iron supplementation

Iron supplementation has benefits [1] and possible harms [2]. Some studies have shown that these benefits are least in subjects who are already relatively replete in iron [eg 3] and that the harms may be greatest in replete subjects [4]. Furthermore, iron frequently induces unpleasant gastrointestinal side effects that limit compliance [5,6]. Finally, iron absorption is least efficient in iron replete subjects [7]. For these reasons, it would be desirable to target iron supplements to subjects who are most at need of iron, will most readily absorb it, and will be least likely to suffer any adverse reactions. Haemoglobin (Hb) levels could provide a field-friendly screening tool, but Hb is a very poor correlate of iron deficiency (ID) because iron-limited erythropoiesis is a late indicator of ID, and because there are multiple other causes of anaemia. Ferritin is another possible indicator but is confounded by inflammation thus requiring assays for both ferritin and an inflammatory marker. We proposed that serum hepcidin level could provide an optimal screening parameter because hepcidin levels reflect a combination of iron status and threat of infection. The rationale for this, described also in our protocol paper [8], is summarised below.

The role of hepcidin – the master regulator of iron metabolism

Iron homeostasis, and its distribution within the body, is maintained by regulating absorption of iron through duodenal enterocytes and by controlling the rate of iron recycling through macrophages. Hepcidin is a small peptide hormone that binds to and causes the degradation of ferroportin, an iron export protein highly expressed by enterocytes and macrophages [9]. High levels of hepcidin thereby inhibit absorption of dietary iron and lock iron in macrophages, rapidly depleting serum iron (thus causing the protective hypoferraemia of the acute phase response) and lowering iron availability for erythropoiesis [7]. An abundance of evidence in humans and experimental animals indicates that the ferroportin-hepcidin interaction is the dominant and non-redundant regulator of iron balance and iron distribution [9]. Regulation of hepcidin is complex – iron accumulation induces hepcidin, providing a negative feedback loop to maintain homeostasis, but hepcidin levels are also increased by inflammatory signals arising during infections [10,11]. Iron deficiency and erythropoietic drive suppress hepcidin, releasing iron from cellular stores and increasing dietary iron absorption. Importantly, each of these signals is variable, and the balance between them determines hepcidin synthesis. Thus, an iron deficient individual may have high serum hepcidin due to an acute infection, but on the other hand severe anaemia may suppress hepcidin even in the presence of inflammation.

Iron redistribution as a component of innate defence against infection

It is widely accepted that the hypoferraemia of the acute phase response represents a highly conserved component of innate defence against a broad-spectrum of extracellular organisms that could elicit a rapidly fatal septicaemia if allowed unrestricted access to circulating iron. Several studies have proposed that iron-requiring intracellular organisms might have evolved their niche specificity precisely to capitalize upon the consequent iron-rich environment in macrophages [eg, 10,12]. Such interactions may play a significant role in explaining susceptibility to secondary infections.

Hepcidin-guided iron supplementation

Because hepcidin reports on the balance of iron status and inflammation, and because hepcidin also determines how well oral iron is absorbed, low hepcidin levels indicate both a requirement for iron and an ability to utilize it if provided. Individuals with high hepcidin may be iron replete, or inflamed, or both, and will not be able to absorb oral iron efficiently. Thus, children with low hepcidin can be described as 'safe-and-ready to receive iron'. We have illustrated this in young children in similar (and the same) settings as the current study, and used ROC analyses to devise a single diagnostic cut-off of 5.5 ng/mL (based on the Bachem ELISA) to identify children suffering from ID and able to absorb iron [13]. We reasoned that providing an extra decision point ('do not give iron unless hepcidin is below a

cut-off level') in iron supplementation programmes should make them both safer and more efficient. This would permit safe iron supplementation of young children in infectious areas allowing them to reach their full human potential.

Together with our previously reported trial in pregnant women [14], these trials represent the first studies to test whether a hepcidin-guided screen-and-treat approach could deliver advantages compared to the standard WHO regimen of universal iron supplementation to all subjects irrespective of iron status or likelihood of infection.

References

1. WHO. Guideline: Use of multiple micronutrient powders for home fortification of foods consumed by infants and children 6–23 months of age. *Geneva, World Heal Organ* 2011; 1–30.
2. Pasricha SR, Armitage AE, Prentice AM, Drakesmith H. Reducing anaemia in low-income countries: control of infection is essential. *BMJ* 2018; 362: k3165.
3. Mwangi MN, Roth JM, Smit MR, Trijsburg L, Mwangi AM, Demir AY, Wielders JP, Mens PF, Verweij JJ, Cox SE, Prentice AM, Brouwer ID, Savelkoul HF, Andang'o PE, Verhoef H. Effect of Daily Antenatal Iron Supplementation on Plasmodium Infection in Kenyan Women: A Randomized Clinical Trial. *JAMA*. 2015; 314: 1009-20.
4. Lynch S, Stoltzfus R, Rawat R. Critical review of strategies to prevent and control iron deficiency in children. *Food Nutr Bull*. 2007; 28(4 Suppl): S610-20.
5. Jaeggi T, Kortman GA, Moretti D, Chassard C, Holding P, Dostal A, Boekhorst J, Timmerman HM, Swinkels DW, Tjalsma H, et al. Iron fortification adversely affects the gut microbiome, increases pathogen abundance and induces intestinal inflammation in Kenyan infants. *Gut* 2015; 64: 731–742.
6. Tolkien Z, Stecher L, Mander AP, Pereira DI, Powell JJ. Ferrous sulfate supplementation causes significant gastrointestinal side-effects in adults: a systematic review and meta-analysis. *PLoS One* 2015; 10: e0117383.
7. Prentice AM, Doherty CP, Abrams SA, Cox SE, Atkinson SH, Verhoef H, Armitage AE, Drakesmith H. Hepcidin is the major predictor of erythrocyte iron incorporation in anemic African children. *Blood.* 2012; 119: 1922–1928.
8. Wegmüller R, Bah A, Kendall L, *et al.* Efficacy and safety of hepcidin-based screen-and-treat approaches using two different doses versus a standard universal approach of iron supplementation in young children in rural Gambia: A double-blind randomised controlled trial. *BMC Pediatr* 2016; 16. DOI:10.1186/s12887-016-0689-4.
9. Ganz T. Hepcidin and iron regulation, 10 years later. *Blood.* 2011; 117: 4425–4433
10. Drakesmith H, Prentice AM. Hepcidin and the iron-infection axis. *Science.* 2012; 338: 768–72.
11. Atkinson SH, Armitage AE, Khandwala S, Mwangi TW, Uyoga S, Bejon PA, Williams TN, Prentice AM, Drakesmith H. Combinatorial effects of malaria season, iron deficiency, and inflammation determine plasma hepcidin concentration in African children. *Blood*. 2014; 123: 3221-9.
12. Ganz T. Iron and infection. *Int J Hematol*. 2018; 107: 7-15.
13. Pasricha SR, Atkinson SH, Armitage AE, Khandwala S, Veenemans J, Cox SE, Eddowes LA, Hayes T, Doherty CP, Demir AY, et al. Expression of the iron hormone hepcidin distinguishes different types of anemia in African children. *Sci Transl Med* 2014; 6: 235re233
14. Bah A, Muhammad AK, Wegmuller R, Verhoef H, Goheen MM, Sanyang S, Danso E, Sise EA, Pasricha SR, Armitage AE, Drakesmith H, Cross JH, Moore SE, Cerami C, Prentice AM. Hepcidin-guided screen-and-treat interventions against iron-deficiency anaemia in pregnancy: a randomised controlled trial in The Gambia. *Lancet Glob Health*. 2019; 7: e1564-e1574.

Laboratory cut-offs

Transferrin: cut-off as in Higgins [1].

Soluble transferrin receptor: >8·3 mg/L, concentrations adjusted to Ramco using Pfeiffer [2].

Erythrocyte glutathione reductase activation coefficient: cut-off as used by IOM derived from McCormick and Greene [3].

Ferritin index: >3·2**.**

Iron: <4·0 μmol/L [4].

Iron deficiency (ID): defined as (ferritin <12μg/L & ferritin index >3·2 if CRP ≤5mg/L) OR (ferritin <30μg/L & ferritin index >2 if CRP >5mg/L).

Iron deficiency anemia (IDA): defined as (Hb <110g/L & ferritin <12μg/L & ferritin index >3·2 if CRP ≤5mg/L) OR (Hb <110g/L & ferritin <30μg/L & ferritin index >2 if CRP >5mg/L).

References

1. Higgins V, Chan MK, Adeli K. Pediatric Reference Intervals for Transferrin Saturation in the CALIPER Cohort of Healthy Children and Adolescents. EJIFCC. 2017; 28: 77-84.
2. Pfeiffer CM, Cook JD, Mei Z, Cogswell ME, Looker AC, Lacher DA. Evaluation of an automated soluble transferrin receptor (sTfR) assay on the Roche Hitachi analyzer and its comparison to two ELISA assays. Clin Chim Acta. 2007; 382: 112-6.
3. McCormick DB, Greene HL. Vitamins. In: Burtis CA, Ashwood ER, editors. Tietz Textbook of Clinical Chemistry. Philadelphia: Saunders; 1994.
4. Parkin PC, Hamid J, Borkhoff CM, et al. Laboratory reference intervals in the assessment of iron status in young children. BMJ Paediatr open 2017; 1: e000074

Supplementary Table 1: Composition of the experimental supplement based upon the MixMe WHO formulation

Micronutrients	Dose/day
Vitamin A (ug RE)	400
Vitamin D (ug)	5
Vitamin E (mg)	5
Vitamin C (mg)	30
Thiamine (B_1) (mg)	0·5
Riboflavin (B_2) (mg)	0·5
Niacin (B_3) (mg)	6
Pyridoxine (B_6) (mg)	0·5
Cobalamine (B_{12}) (ug)	0·9
Folate (ug)	150
Iron (encapsulated ferrous fumarate) (mg)	12 or 6 or 0
Zinc (mg)	4·1
Copper (mg)	0·56
Selenium (ug)	17
Iodine (ug)	90

Supplementary Table 2: Additional baseline characteristics of the intention-to-treat population

Characteristics	Reference group (n=135)	12 mg screen-and-treat group (n=136)	6 mg screen-and-treat group (n=136)
**Anthropometry**			
Height, cm	75·8 (5·0)	75·7 (4·7)	75·6 (4·9)
Weight, kg	8·9 (1·2)	8·8 (1·2)	8·7 (1·3)
Head circumference, cm	45·1 (1·8)	44·8 (1·7)	45·0 (1·7)
Mid-upper arm circumference (MUAC), cm	14·3 (0·8)	14·3 (0·9)	14·2 (0·9)
Triceps skinfold thickness, cm	7·1 (1·2)	7·0 (1·2)	6·9 (1·1)
Height-for-age z-score (HAZ)	-0·97 (0·93)	-1·00 (0·90)	-1·05 (0·93)
Stunted (HAZ<-2)	18/135 (13%)	17/136 (13%)	23/136 (17%)
Weight-for-age z-score (WAZ)	-1·08 (0·85)	-1·15 (0·93)	-1·29 (0·93)
Underweight (WAZ<-2)	17/135 (13%)	27/136 (20%)	35/136 (26%)
Weight-for-height z-score (WHZ)	-0·80 (0·83)	-0·90 (0·94)	-1·06 (0·95)
Wasted (WHZ<-2)	12/135 (9%)	14/136 (10%)	25/136 (18%)
Head circumference-for-age z-score	-0·75 (0·90)	-0·94 (0·96)	-0·85 (0·96)
MUAC-for-age z-score	-0·23 (0·70)	-0·26 (0·77)	-0·34 (0·72)
Triceps skinfold-for-age z-score	-0·62 (0·91)	-0·73 (0·87)	-0·79 (0·87)
**Haematology**			
Haemoglobin concentration, g/L (by HemoCue 301 field photometer)	101 (93, 105)	102 (95, 106)	100 (94, 105)
Anaemia (haemoglobin <110 g/L) (by HemoCue 301 field photometer)	135/135 (100%)	136/136 (100%)	135/136 (99%)
Mean corpuscular volume (MCV), fL	62·8 (6·9)	63·2 (7·1)	62·3 (7·4)
Mean corpuscular haemoglobin (MCH), pg	22·2 (2·7)	22·2 (3·0)	21·9 (2·9)
Mean corpuscular haemoglobin concentration (MCHC), g/dL	35·3 (1·3)	35·1 (1·4)	35·2 (1·5)
Red blood cell distribution width, %	17·8 (1·1)	17·5 (1·2)	17·5 (1·1)
White blood cell count, $x10^9$/L	11·5 (9·2-13·7)	11·4 (9·2-13·7)	11·8 (9·3-14·2)
**Plasma iron markers**			
Ferritin if CRP<5 mg/L, μg/L	11·1 (5·9-18·6)	11·1 (4·2-21·5)	9·2 (2·2-18)
Iron <4·0 μmol/L	52/121 (43%)	47/117 (40%)	43/112 (38%)
Iron deficient erythropoiesis (sTfR > 8·3 mg/L)	112/125 (90%)	113/127 (89%)	114/133 (86%)
sTfR/log ferritin ratio (ferritin index)	5·2 (3·1-8·5)	5·3 (3·5-8·6)	5·3 (3·1-12·5)
Ferritin index > 3·2 [a]	89/122 (73%)	93/118 (79%)	93/124 (75%)
**Inflammation markers**			
CRP >5·0 mg/L OR AGP >1·0 g/L	83/126 (66%)	85/126 (67%)	92/133 (69%)
Current or recent *P· falciparum* infection	0/135 (0%)	0/136 (0%)	0/136 (0%)

Data are mean (SD), n/N (%), or median (IQR). AGP=α1-acid glycoprotein; CRP=C-reactive protein; sTfR=soluble transferrin receptor. [a] children with CRP>5 excluded

Supplementary Table 3: Impact of screen-and-treat design on iron intakes (per-protocol analysis)

	Number of participants/total with data available (n/N)	Prevalence (%)	Effect (95% CI)
**Ready-and-safe-to receive iron (hepcidin<5·5 µg/L)**			
Reference group	32/115	28%	
12 mg screen-and-treat group	43/114	38%	9·9 (-2·2, 22·0)
6 mg screen-and-treat group	51/112	46%	17·7 (5·4, 30·0)
	N	Days	Effect (95% CI)
**Iron prescribed (number of days in which iron-containing MMPs were prescribed)[1]**			
Reference group	115	83 (83-83)	
12 mg screen-and-treat group	116	42 (0-83)	-41·7 (-47·1, -36·3)
6 mg screen-and-treat group	114	41 (0-83)	-42·3 (-47·7, -36·9)
**Adherence (number of days in which iron-containing MMPs were consumed)[1]**			
Reference group	115	82 (75-83)	
12 mg screen-and-treat group	116	41·5 (0-83)	-40·9 (-46·3, -35·6)
6 mg screen-and-treat group	114	40·5 (0-83)	-41·6 (-47·0, -36·3)
	N	Average dose (mg)	Effect (95% CI)
**Dose of iron received over whole study period, mg[1]**			
Reference group	115	984 (900-996)	
12 mg screen-and-treat group	116	498 (0-996)	-491 (-540, -441)
6 mg screen-and-treat group	114	243 (0-498)	-740 (-790, -690)

[1] median (range). Prevalence = proportion of total as %.

Supplementary Table 4: Secondary haematological outcomes, continuous variables at Day 84 (per-protocol analysis) *

	Participants with data available n (% of randomized)	Estimate (95% CI)	Effect (95% CI)
**Haemoglobin concentration (by HemoCue field photometer)**			
Reference group	115 (85·2)	109·4 g/L (107·6, 111·2 g/L)	
12 mg screen-and-treat group	116 (85·3)	107·3 g/L (105·7, 108·8 g/L)	-2·1 g/L (-4·5, 0·2 g/L)
6 mg screen-and-treat group	114 (83·8)	105·3 g/L (103·4, 107·1 g/L)	-4·2 g/L (-6·8, -1·5 g/L)
**Haematocrit**			
Reference group	115 (85·2)	29·7% (28·8, 30·7%)	
12 mg screen-and-treat group	116 (85·3)	28·2% (27·7, 28·7%)	-1·5% (-2·6, -0·4%)
6 mg screen-and-treat group	114 (83·8)	27·5% (27·0, 28·0%)	-2·3% (-3·3, -1·2%)
**Red blood cell distribution width**			
Reference group	115 (85·2)	17·8% (17·4, 18·2%)	
12 mg screen-and-treat group	116 (85·3)	18·2% (17·7, 18·6%)	2·3% (-1·1, 5·8%)
6 mg screen-and-treat group	114 (83·8)	17·9% (17·4, 18·4%)	0·8% (-2·7, 4·4%)
**Mean corpuscular volume**			
Reference group	115 (85·2)	65·9 fL (64·8, 66·9 fL)	
12 mg screen-and-treat group	116 (85·3)	64·7 fL (63·5, 65·9 fL)	-1·1 fL (-2·8, 0·5 fL)
6 mg screen-and-treat group	114 (83·8)	62·9 fL (61·6, 64·1 fL)	-3·0 fL (-4·6, -1·4 fL)
**Mean corpuscular haemoglobin**			
Reference group	115 (85·2)	23·2 pg (22·8, 23·6 pg)	
12 mg screen-and-treat group	116 (85·3)	22·7 pg (22·2, 23·2 pg)	-0·5 pg (-1·2, 0·1 pg)
6 mg screen-and-treat group	114 (83·8)	22·2 pg (21·7, 22·6 pg)	-1·1 pg (-1·7, -0·5 pg)
**Mean corpuscular haemoglobin concentration**			
Reference group	115 (85·2)	35·3 g/L (35·0, 35·5 g/L)	
12 mg screen-and-treat group	116 (85·3)	35·1 g/L (34·8, 35·3 g/L)	-0·2 g/L (-0·5, 0·1 g/L)
6 mg screen-and-treat group	114 (83·8)	35·2 g/L (35·0, 35·4 g/L)	-0·05 g/L (-0·4, 0·3 g/L)
**White blood cell count**			

Reference group	115 (85·2)	11·3 x10^9/L (10·6, 12·0) x10^9/L	
12 mg screen-and-treat group	116 (85·3)	11·8 x10^9/L (11·2, 12·3 x10^9/L)	4·3% (-3·4, 12·5%)
6 mg screen-and-treat group	113 (83·1)	11·9 x10^9/L (11·3, 12·6 x10^9/L)	5·7% (-2·6, 14·7%)

[*] Mixed effects linear regression with study group as fixed effect and participant as random effect factor; log transformed data for red blood cell distribution width, and white blood cell count; all log transformed data were exponentiated for presentation of estimates as geometric mean in the table; effect sizes are unadjusted and are presented as absolute effects for non-transformed variables and as relative effects (percentage change in geometric mean relative to the reference group) for log transformed variables.

Supplementary Table 5: Safety outcomes

	**Observed number of events**	**OR (95% CI)**
**Twice weekly morbidity monitoring**		
Fever		
Reference	8	
12 mg screen-and-treat	10	0.77 (0.03, 20.9)
6 mg screen-and-treat	10	0.58 (0.02, 16.75)
Diarrhoea		
Reference	8	
12 mg screen-and-treat	3	0.02 (0.00, 4.2)
6 mg screen-and-treat	8	0.30 (0.01, 7.99)
Vomiting		
Reference	2	
12 mg screen-and-treat	1	0.33 (0.03, 3.89)
6 mg screen-and-treat	1	0.31 (0.03, 3.744)
Cough		
Reference	4	
12 mg screen-and-treat	3	0.47 (0.91, 2.39)
6 mg screen-and-treat	3	0.45 (0.88, 2.29)
**All adverse events***		
Diarrhoea		
Reference	40	
12 mg screen-and-treat	49	1.26 (0.75, 2.14)
6 mg screen-and-treat	63	1.69 (1.02, 2.83)
Skin infection		
Reference	38	
12 mg screen-and-treat	38	0.95 (0.57, 1.57)
6 mg screen-and-treat	46	1.14 (0.70, 1.85)
Cough		
Reference	65	
12 mg screen-and-treat	71	1.08 (0.69, 1.69)
6 mg screen-and-treat	58	0.74 (0.47, 1.17)
Pneumonia		
Reference	25	
12 mg screen-and-treat	18	0.66 (0.35, 1.25)
6 mg screen-and-treat	19	0.66 (0.35, 1.25)
Malaria		
Reference	2	
12 mg screen-and-treat	1	0.48 (0.04, 5.30)
6 mg screen-and-treat	1	0.46 (0.04, 5.06)

* includes 8 serious adverse events

Supplementary Figure 1: Schematic summary of cohort timing

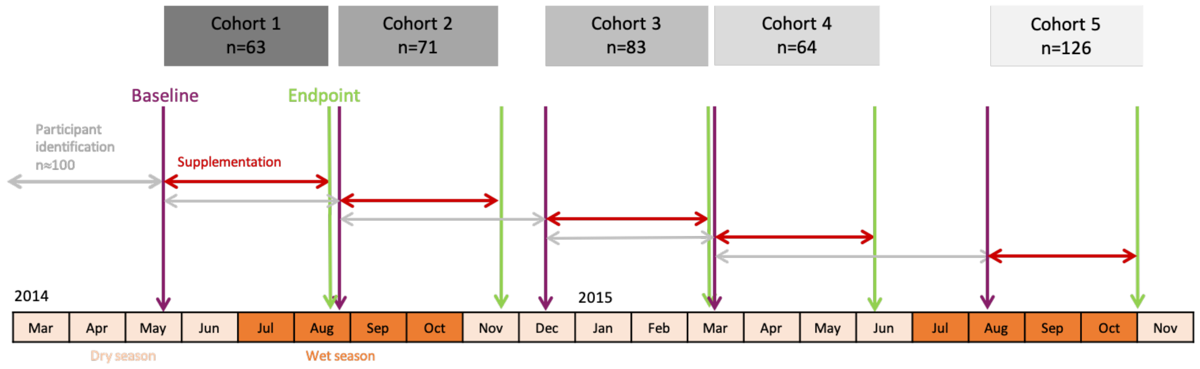

Supplementary Figure 2: Trial design and timelines

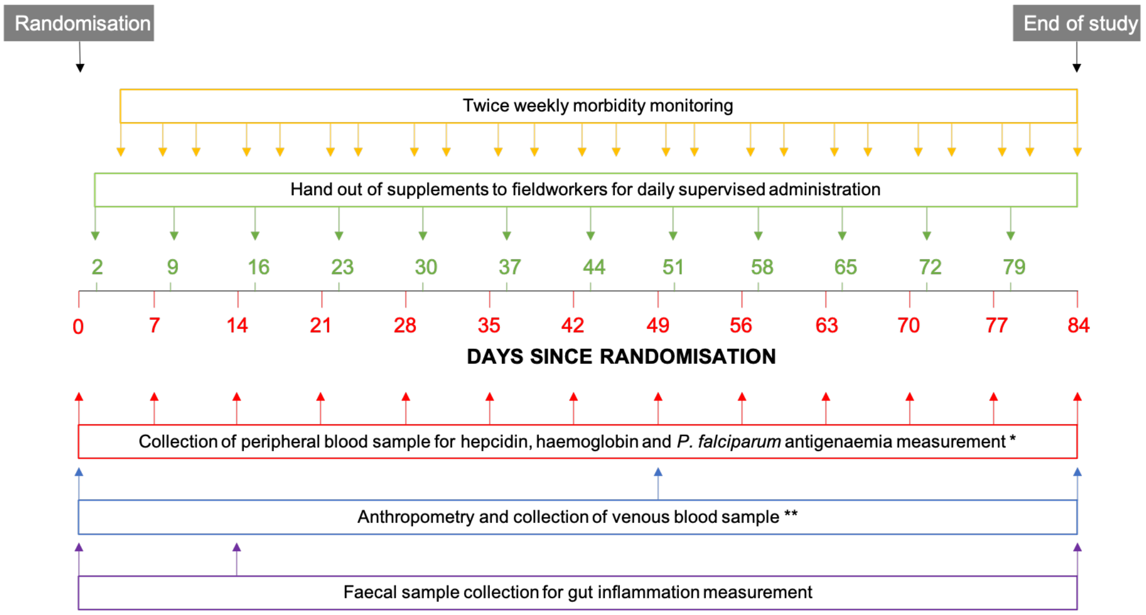

*Haemoglobin concentration was determined by HemoCue and *P. falciparum* antigenaemia by rapid diagnostic test

**For haemogram by automated blood analyser and to determine concentrations of iron markers, inflammatory markers, malaria parasite growth in RBCs and bacterial growth in plasma

CLINICAL STUDY PROTOCOL

Title

Efficacy and safety of hepcidin-based screen-and-treat approaches using two different doses vs a standard universal approach of iron supplementation in young children in rural Gambia: a double-blind randomized controlled trial.

**Protocol No:**	**SCC 1358**
**Brief Title:**	Hepcidin and anaemia in young children
**Protocol Version**	3.0 – 21 July 2014
**Sponsor**	MRC International Nutrition Group,<br>MRC Keneba,<br>PO Box 273 Banjul,<br>The Gambia
**Principal Investigator**	Dr Rita Wegmüller<br><br>MRC International Nutrition Group,<br>MRC Keneba,<br>PO Box 273 Banjul,<br>The Gambia

Protocol amendment(s)

Amendment #: 2

Principal Investigator:	Signature:	Date:
Rita Wegmüller, PhD<br>Name, Title		21/07/2014
**Sponsor's representative:**	**Signature:**	**Date:**
Andrew Prentice, Prof.<br>Name, Title		21/07/2014

Signature page

The study will be carried out in accordance with the protocol, the principles of good clinical practice and in accordance to local legal and regulatory requirements.

**Principal Investigator:**	**Signature:**	**Date:**
Rita Wegmüller<br>*Name, Title*	R. Wegmüller	12/12/2013

**Sponsor's representative:**	**Signature:**	**Date:**
Andrew Prentice<br>*Name, Title*	AM Prentice	12/12/2013

Table of contents

Page
Protocol amendment(s) 14
Signature page 15
Key roles 18
List of abbreviations 20
Protocol summary 21
1 Background information and rationale 23
1.1 Background information 23
1.1.1 Iron deficiency: global prevalence, health consequences, and barriers to progress in elimination 23
1.1.2 Evidence for detrimental effects of iron 23
1.1.3 The role of Hepcidin – the master regulator of iron metabolism 24
1.1.4 Iron redistribution as a component of innate defence against infection 24
1.1.5 Hepcidin-guided iron supplementation 24
1.1.6 Multiple micronutrient powders 25
1.1.7 Rationale 25
1.2 Potential risks and benefits 26
2 Study objectives 26
2.1 Study endpoints 26
3 Study design 27
3.1 Type of study and design 27
3.2 Randomisation and blinding procedures 28
3.2.1 Randomisation 28
3.2.2 Blinding 29
3.3 Sub-studies 29
3.4 Investigational products or interventions 30
3.4.1 Description of product or intervention 30
3.4.2 Formulation, packaging and labelling 30
3.4.3 Product storage and stability 31
3.5 Dosage, preparation and administration of investigational product or intervention 31
3.6 Concomitant medications/treatments 32
4 Selection and withdrawal of participants 32
4.1 Selection of participants 32
4.2 Eligibility of participants 32
4.2.1 Inclusion criteria 32
4.2.2 Exclusion criteria 32
4.3 Withdrawal of participants 33
5 Study procedures and evaluations 33
5.1 Study schedule 33
5.1.1 Study sensitisation 33
5.1.2 Screening and enrollment (Baseline) 33
5.1.3 Follow-up 34
5.1.4 Final study visit 35
5.1.5 Early termination visit 35
5.2 Study evaluations 35

5.2.1 Clinical evaluations 36
5.2.2 Laboratory evaluations 36
6 Safety considerations 37
6.1 Methods and timing for assessing, recording, and analysing safety parameters 37
6.1.1 Adverse events 37
6.1.2 Serious adverse events (SAEs) 37
6.1.3 Assessment of intensity 37
6.1.4 Assessment of causality 38
6.2 Reporting procedures 39
6.3 Safety oversight 40
7 Discontinuation criteria 40
7.1 Participant's premature termination 40
7.2 Study discontinuation 40
8 Statistical considerations 40
8.1 Sample size determination 40
8.2 Statistical analysis 41
9 Data handling and record keeping 42
9.1 Data management and processing 42
9.2 Source documents and access to source data 42
9.3 Protocol deviations 42
10 Quality control and quality assurance 43
10.1 Study monitoring 43
11 Ethical considerations 43
11.1 General considerations on human subject protection 43
11.1.1 Rationale for participant selection 43
11.1.2 Evaluation of risks and benefits 44
11.2 Informed consent 44
11.3 Participant confidentiality 44
11.4 Future use of stored specimen 45
12 Financing and insurance 45
13 Publication policy 45
14 References 45
Appendix 1: Schematic of study design - main study 47
Appendix 2: Schematic of study design – sub-study 48
Appendix 3: Schedule of events 49
Appendix 4: Declaration of Helsinki 50

Key roles

For questions regarding this protocol, contact Dr Rita Wegmüller at MRC Keneba rwegmueller@mrc.gm; tel: 002209963991

**Author:**	Rita Wegmüller, PhD<br>MRC International Nutrition Group,<br>MRC Keneba, PO Box 273 Banjul,<br>The Gambia<br>Phone: 0022099639916;<br>E-mail: rwegmuller@mrc.gm
**Sponsor's representative:**	Prof Andrew Prentice
**Trial Physician:**	Dr Patrick Nshe
**Trial monitor(s):**	Mr Edrisa Sinjanka<br>MRC International Nutrition Group, MRC Keneba,<br>PO Box 273 Banjul, The Gambia<br>Phone: 002209952135<br>E-mail: esinjanka@mrc.gm
**Chair of DMC/DMSB:**	Dr James Jay Berkley<br>KEMRI Centre for Geographic Medicine Research<br>KEMRI-Wellcome Trust Research Programme<br>Kilifi, Kenya
**Data Manager:**	Mr Mohammed Ngum<br>MRC International Nutrition Group, MRC Keneba, PO Box 273 Banjul, The Gambia<br>Phone: 002207662013; E-mail: mngum@mrc.gm
**Statistician:**	Ms Lindsay Kendall<br>MRC Unit The Gambia, PO Box 273, Banjul, The Gambia<br>E-mail: lkendall@mrc.gm

**Clinical Laboratory/ies:**	WIMM, University of Oxford, United Kingdom<br>University of North Carolina, USA<br>ETH Zurich, Switzerland
**Other institutions:**	National Nutrition Agency [NaNA] and the Ministry of Health
**Ethics Committee**	Gambia Government/MRC Joint Ethics Committee,<br>c/o MRC Unit, The Gambia,<br>PO Box 273, Banjul, The Gambia, West Africa

List of abbreviations

AE	Adverse Event
AGP	Alpha-1 acid Glycoprotein
CRF	Case Report Form
CRP	C-reactive protein
DSM	Dutch State Mines
DSMB	Data Safety Monitoring Board
GCP	Good Clinical Practice
GMP	Good Manufacturing Practice
Hb	Haemoglobin
ID	Iron deficiency
IDA	Iron Deficiency Anaemia
IEC	Independent Ethics Committee
ITM	Independent Trial Monitor
MNP	Micronutrient Powder
MRC	Medical Research Council
MRC ING	Medical Research Council International Nutrition Group
PI	Principal Investigator
PoC	Point of Care
RCH	Reproductive and Child Health
RCT	Randomised Controlled Trial
RDT	Rapid Diagnostic Test
RHT	Regional Health Team
SCC	Scientific Coordinating Committee
SF	Serum Ferritin
sTfR	Soluble Transferrin Receptor
TSAT	Transferrin Saturation
WHO	World Health Organisation
WIMM	Weatherall Institute of Molecular Medicine
ZnPP	Zinc Protoporphyrin

Protocol summary

**Title:**	Efficacy and safety of hepcidin-based screen-and-treat approaches using two different doses vs a standard universal approach of iron supplementation in young children in rural Gambia: a double-blind randomized controlled trial.
**Brief title:**	Hepcidin and anaemia in young children
**Population:**	Healthy children, 6 – 24 months old
**Number of participants:**	393 young children
**Number of Sites:**	12 communities of Soma, Karantaba, Kani Kunda, Sankwia, Mansakonko, Pakalinding, Jenoi, Si Kunda, Toniataba, Jiffin, Kaiaf and Genieri
**Location of Sites:**	Jarra West and Kiang East located in the Lower River Region
**Study Duration:**	
**- Clinical Phase:**	1.5 years
**- Whole study:**	2 years
**Duration for Participants:**	12 weeks<br>15 months for the 90 children also included in the sub-study (intervention of 12 weeks as in main study)
**Description of Products or Intervention:**	Micronutrient powder (MixMe) containing 12 mg, 6 mg or 0 mg iron per sachet (dose)
**Objectives:**	*Primary objective:*<br>To evaluate whether equivalent efficacy against iron deficiency (ID) and Iron Deficiency Anaemia (IDA) can be achieved using screen-and-treat* at 12 mg iron/day or screen-and-treat at 6 mg iron/day compared to 12 mg iron/day universal supplementation.<br>*Secondary objective:*<br>a) To evaluate the feasibility of adopting a hepcidin-based screen-and-treat approach to iron supplementation in young children<br>b) To evaluate whether a screen-and-treat approach is beneficial with respect to:<br>• Maternal/guardian reporting of illnesses<br>• Safety indices (inflammatory and immune activation markers, faecal calprotectin and zonulin, gut microbiota, *ex-vivo* bacterial and *P. falciparum* growth)

c) To evaluate overall iron absorption over the 12 weeks supplementation period in the 3 study groups (sub-study)

*screen-and-treat refers to provision of the next week's iron or placebo (MNP without iron) MNP being conditional on the subject having a hepcidin value below or above 5.5 ng/mL

Description of Study Design:

Proof-of-concept, 3-arm, double blind, randomised controlled trial (RCT) in young children. The young children will be randomly assigned in a 1:1:1 ratio to either receive a daily sachet of MNP for 12 weeks of:

Group A: MNP containing 12 mg iron/day (n = 131);

Group B: MNP containing 12 mg iron/day if hepcidin is below 5.5 ng/mL or MNP containing 0 mg iron/day if hepcidin is above 5.5 ng/mL based on a weekly hepcidin screening indicating if iron can be given for the next 7 days or not (n = 131);

Group C: MNP containing 6 mg iron/day if hepcidin is below 5.5 ng/mL or MNP containing 0 mg iron/day if hepcidin is above 5.5 ng/mL based on a weekly hepcidin screening indicating if iron can be given for the next 7 days or not (n = 131).

1 Background information and rationale

1.1 Background information

1.1.1 Iron deficiency: global prevalence, health consequences, and barriers to progress in elimination

Iron deficiency (ID), leading to iron deficiency anaemia (IDA) and impaired neurocognitive development, remains the most pervasive nutritional deficiency worldwide. Using currently accepted criteria the prevalence rates for IDA in young children frequently exceed 50% in low-income countries (as is the case in The Gambia) resulting in impaired immunocompetence and brain development and thus leading to substantial loss of human potential[1]. Low cost iron supplements are efficacious in combatting IDA so, in countries with anaemia rates of >40%, WHO recommended universal supplementation of pregnant women and young children. In 2006, the Pemba Trial was prematurely terminated due to significant increases in the number of serious adverse outcomes and deaths in young children receiving iron-folate supplements[2]. This result was attributed to a malign interaction between iron and malaria (since a parallel trial in a non-malarious area of Nepal had revealed no increase in adverse outcomes), and WHO revised its policy guidance for malarious regions[3]. The new guidelines recommended adoption of a screen-and-treat approach or the use of centralized or point-of-use food fortification using micronutrient powders which was considered likely to be a safer option. However, recent studies, including a very large trial from a non-malarious area in Pakistan[4], have revealed important evidence of medically significant adverse outcomes associated with iron administration. It is assumed that all of these adverse outcomes may be attributable to host-pathogen competition for iron whereby supplemental iron has favoured pathogens more rapidly than their host. Thus there is an urgent need: a) to understand the pathways by which provision of iron favours pathogen virulence; and b) to use this knowledge to design safer modes for preventative and therapeutic provision of iron to infants and young children living in infectious environments in order to make them more resistant against infections and to allow maximal brain development.

1.1.2 Evidence for detrimental effects of iron

Iron is unique among nutrients in being both essential and highly toxic. This tension has driven the evolution in humans of complex systems for regulating iron absorption, and safely chaperoning it during transport, storage and utilization[5]. The discovery of the iron-regulatory hormone hepcidin has thrown a sharp new focus on the adaptive/protective value of maintaining strict physiological control over iron absorption.

Initially the Pemba results were viewed with some scepticism in many quarters and/or were assumed to only affect malarious regions, as well as being confined to supplementation as opposed to fortification. Additional intervention trials have confirmed that administration of iron, either alone or as part of multiple micronutrient formulations, can lead to serious adverse effects as follows: a) a second trial in Tanzania confirmed that micronutrient supplements containing iron increase the risk of malaria[6]; b) a trial of iron-fortified biscuits given to children in Cote d'Ivoire caused significant adverse re-profiling of the gut microflora and evidence of intestinal inflammation[7]; c) a large cluster-randomized trial of iron-containing Sprinkles in 17,000+ Pakistani children has revealed significant increases in diarrhoea and pneumonia rates and a very significant increase in severe and bloody diarrhea[4]. However, a recently published trial in young Ghanaian children receiving multiple micronutrient powders showed a trend towards lower malaria rates in the group receiving iron but an increase in hospitalisations among the iron group[8].

1.1.3 The role of Hepcidin – the master regulator of iron metabolism

Iron homeostasis, and its distribution within the body, is maintained by regulating absorption of iron through duodenal enterocytes and by controlling the rate of iron recycling through macrophages. Hepcidin is a small peptide hormone that binds to and causes the degradation of ferroportin, an iron export protein highly expressed by enterocytes and macrophages[9]. High levels of hepcidin thereby inhibit absorption of dietary iron and lock iron in macrophages, rapidly depleting serum iron (causing the protective hypoferraemia of the acute phase response) and lowering iron availability for erythropoiesis[10]. An abundance of genetic evidence in humans and experimental animals indicates that the ferroportin-hepcidin interaction is the dominant and non-redundant regulator of iron balance and iron distribution. Regulation of hepcidin is complex – iron accumulation induces hepcidin, providing a negative feedback loop to maintain homeostasis, but hepcidin levels are also increased by inflammatory signals arising during infections. Iron deficiency suppresses hepcidin, releasing iron from stores and increasing iron absorption. Importantly, each of these signals is variable, and the balance between them determines hepcidin synthesis. Thus, an iron deficient individual may have high serum hepcidin due to an acute infection, but on the other hand severe anaemia may suppress hepcidin even in the presence of inflammation.

1.1.4 Iron redistribution as a component of innate defence against infection

It is widely accepted that the hypoferraemia of the acute phase response represents a highly conserved component of innate defence against a broad-spectrum of extracellular organisms that could elicit a rapidly fatal septicaemia if allowed unrestricted access to circulating iron. We and others have proposed that iron-requiring intracellular organisms might have evolved their niche specificity precisely to capitalize upon the consequent iron-rich environment in macrophages[11]. Such interactions may play a significant role in explaining susceptibility to secondary infections. Experimental validation in animal and human studies may have important therapeutic implications.

1.1.5 Hepcidin-guided iron supplementation

Because hepcidin reports on the balance of iron status and inflammation, and because hepcidin also determines how well oral iron is absorbed[9], low hepcidin levels indicate both a requirement for iron and an ability to utilize it if provided. Individuals with high hepcidin may be iron replete, or inflamed, or both, but will not be able to absorb oral iron efficiently. In our paper submitted to Science Translational Medicine this is clearly illustrated in young children and our results suggest a cut-off of 5.5 ng/mL (Figure 1). As discussed above, iron supplementation is associated with deleterious consequences if it is not absorbed. Therefore, providing an extra decision point (do not give iron unless hepcidin is below a cut-off level) in iron supplementation programs should make them both safer and more efficient. This will allow safe iron supplementation of young children in infectious areas allowing them to reach their full human potential.

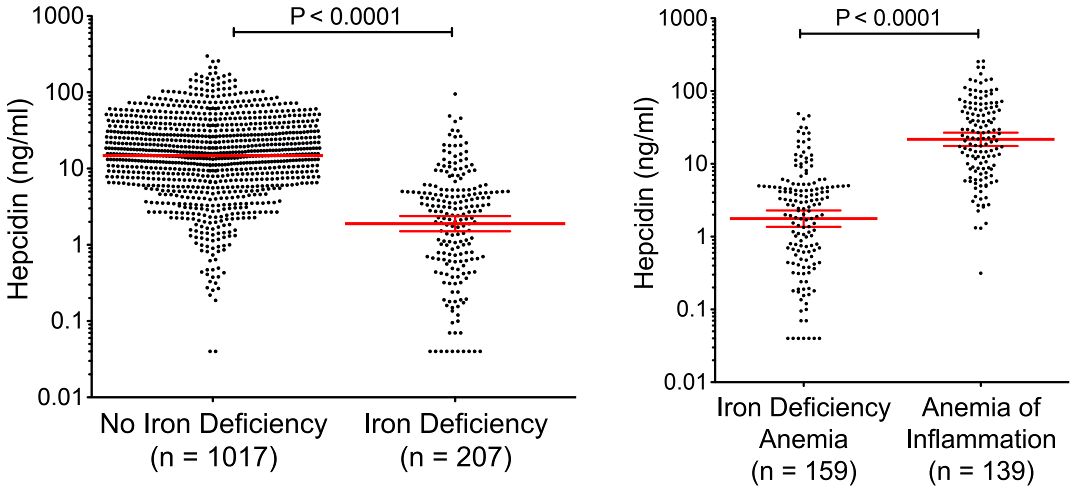

Figure 1: Performance of hepcidin to indicate iron deficiency and distinguish iron deficiency anaemia from anaemia of inflammation

1.1.6 Multiple micronutrient powders

In this trial we will use a micronutrient powder (MixMe) as it is used by UNICEF and WFP. In a Cochrane systematic review (including 8 large trials from a variety of settings, including malaria-endemic areas) assessing the effects and safety of home fortification with multiple micronutrient powders of foods consumed by children under 2 years showed that anaemia was reduced by 31% and iron deficiency by 51%[12]. The review found no difference in the effect of the intervention among children living in malaria endemic areas or areas with sporadic malarial cases. Based on this review WHO guidelines on home fortification were developed and strongly recommend home fortification with MNP to improve iron status and reduce anaemia among infants and young children 6-23 months of age[13].

More recently two other trials investigating the effect of MNP, one in Ghana and one in Pakistan, have been published. The trial in Pakistan showed significant increases in diarrhoea and bloody diarrhoea and pneumonia rates[4], whereas the trial in Ghana showed a trend towards lower malaria rates in the group receiving iron but an increase in hospitalisations among the iron group[8]. Although our trial will not be powered to assess morbidity we will monitor our children very closely and assess morbidity twice weekly.

1.1.7 Rationale

Prevalence of anaemia is > 50% and remains a major public health problem in young children in the Gambia. Combating anaemia due to iron deficiency is a challenge due to the potential negative side-effects of iron when given to people with infections, as is common in The Gambia. The present study of screen-and-treat aims to minimise exposure to iron in too high quantities and at the wrong time and should allow maximising the absorption and utilisation of iron when it is most needed. If this screen-and-treat approach using a hepcidin cut-off to define 'ready and safe to give iron' shows equal efficacy to universal supplementation this approach could be used in health centres. The development of a PoC (point of care) test which would immediately tell the nurse/physician if the person is ready to receive iron would make iron administration more targeted and safer. MRC ING is developing plans with Intrinsic Life Sciences in San Diego to develop such a test.

1.2 Potential risks and benefits

The potential risks to human subjects and known benefits, if any, are summarised in Section "Ethical consideration", Section 11.

2 Study objectives

Primary objective:

To evaluate whether equivalent efficacy against iron deficiency (ID) and Iron Deficiency Anaemia (IDA) can be achieved using screen-and-treat at 12 mg iron/day or screen-and-treat at 6 mg iron/day compared to 12 mg iron/day universal supplementation.

Secondary objective:

a. To evaluate the feasibility of adopting a hepcidin-based screen-and-treat approach to iron supplementation in young children
b. To evaluate whether a screen-and-treat approach is beneficial with respect to:
    - Maternal/guardian reporting of illnesses
    - Safety indices (inflammatory and immune activation markers, faecal calprotectin and zonulin, gut microbiota, *ex-vivo* bacterial and *P. falciparum* growth)
c. To evaluate overall iron absorption over the 12 weeks supplementation period in the 3 study groups (sub-study)

2.1 Study endpoints

Primary endpoint:

Haemoglobin concentration at study day 84 (end of supplementation period) (related to primary objective)

Secondary endpoints:

a) Related to primary objective:
    i. Proportion of anaemia (Hb < 11 g/dL) at study day 84
    ii. Proportion of iron deficiency (sTfR/logFerritin ratio <2.0 ; hepcidin < 5.5 ng/L) at day 84
    iii. Proportion of iron deficiency anaemia (Hb < 11 g/dL & sTfR/logFerritin ratio < 2.0 and ferritin < 12 ug/L or < 30 ug/L in the presence of inflammation) at day 84
b) Related to secondary objective a.:
    i. Iron dosage (number of weeks supplemented)
c) Related to secondary objective b.:
    i. Morbidity (as reported by mothers)
    ii. Safety (inflammatory and immune activation markers, faecal calprotectin and zonulin, gut microbiota, *ex-vivo* bacterial and *P. falciparum* growth)
d) Related to secondary objective c.:

i. Amount of iron absorbed over the supplementation period (Day 0 to 84) (sub-study)

ii. Amount of iron absorbed over the control period (Day 84 to 168) (sub-study)

3 Study design

For a schematic of study design, see Appendix 1.

3.1 Type of study and design

Proof-of-concept, 3-arm, double blind, randomised controlled trial (RCT) over a period of 12 weeks, comparing in parallel groups:

A. Supplementation with a micronutrient powder (MNP) containing 12 mg iron daily (reference treatment);

B. Supplementation with a MNP containing 12 mg iron/day if hepcidin is below 5.5 ng/mL or MNP containing 0 mg iron/day if hepcidin is above 5.5 ng/mL based on a weekly hepcidin screening indicating if iron can be given for the next 7 days or not;

C. Supplementation with a MNP containing 6 mg iron/day if hepcidin is below 5.5 ng/mL or MNP containing 0 mg iron/day if hepcidin is above 5.5 ng/mL based on a weekly hepcidin screening indicating if iron can be given for the next 7 days or not.

Young children in the participating communities will be identified using immunization records at the respective health centres. Once mothers/guardians of the child have signed the informed consent form, the child will be physically examined, height and weight will be measured and a finger prick blood sample for Hb and RDT testing will be taken if all other inclusion criteria and no exclusion criteria are fulfilled. If Hb is ≥7 and < 11 g/dL and the RDT is negative a venous blood sample (5 mL) will be collected. We will then also collect a stool sample from the child. Enrolled children will be randomized (balanced randomization according to Hb and age) to one of the three study arms and will start supplementation two days later when results from hepcidin analysis are available.

Trained field workers will be visiting all children daily during the 12 weeks supplementation period in order to supervise the MNP administration and to check on the children's health status. Twice weekly, morbidity data will be captured. If a child is found unwell, the study nurse will check on the child and decide on treatment/referral to the next health centre. Every week children will be screened using a fingerprick blood sample (200 µL) to determine their hepcidin, Hb and malaria status. Hb and RDT testing will be directly conducted in the field whereas hepcidin analysis will be performed in the laboratory in Keneba. When hepcidin results are available on the next day the respective MNP for the next 7 days for each child will be provided to the field assistants.

On Days 14 and 84 another stool sample and on Days 49 and 84 another venous blood sample (5 mL) instead of a finger prick blood sample will be collected.

Study participants will be recruited in 4 cohorts (12 weeks of supplementation in each cohort) in order to spread the study over a full calendar year and thus take into account seasonality. The plan is to start with a first cohort of about 100 participants (about 1/4 of the total number of 393 participants to be recruited) equally randomized to the 3 study

arms in February 2014. The second cohort will be started in May, the third in August and the last in November 2014. Thus, we will finish study enrolment within approximately 1 year.

Participation of a study subject may be terminated in case the participant withdraws consent, becomes severely anaemic or presents with another exclusion criteria or a SAE or is lost to follow-up.

3.2 Randomisation and blinding procedures

3.2.1 Randomisation

Recruited children will be randomly assigned (computer generated) to one of the 3 treatment arms (equal number in each arm) balanced by the Hb concentration of the baseline blood sample and age. This will assure that Hb concentration in the 3 treatment arms will not differ at baseline. At each day of recruitment (≈ 25 children/day from 1 cluster), each subject will be categorised into two Hb classes (above and below the median Hb of the respective day). Further, children will also be categorized according to age into 3 classes (6-11 months, 12-17 months, 18-24 months). This will divide the children into 6 different classes. In each of the classes children will be randomly assigned to the 3 treatment arms using block randomization.

The randomization will be set up as follows:

i. List of subjects for the respective day of recruitment (n≈25 children)

ii. Subjects categorise into 2 Hb classes (above and below the median of the day)

iii. Subjects categorise into 3 age classes (6-11 mo, 12-17 mo, 18-24mo)

iv. Children in each of the 6 classes listed according to a random number allocated (between 0 and 1)

    a. Low Hb, young age
    b. Low Hb, middle age
    c. Low Hb, old age
    d. High Hb, young age
    e. High Hb, middle age
    f. High Hb, old age

v. Children randomized (using a predetermined block randomization) according to the random number allocated above for each class.

The database to randomize into treatment arms (A, B, C) will be password protected with only the developer knowing the password. After allocation safety envelopes will be produced (1 for each subject) with the subject ID on the front and the treatment allocation in the envelope. The independent trial monitor will be the only person who has access to these envelopes. Thus, if a subject needs to be unblinded at the request of the DSMB their treatment can be easily identified without unblinding the whole study.

3.2.2 Blinding

Participants, field workers (Senior Field Assistants, Field Assistants, Field Supervisor and Field Coordinator), study nurse and PI will be blinded as to which treatment group participants belong to and which supplement participants receive each week. The MNP sachets will be pre-packed on a weekly base by the Field Coordinator in Keneba using lists automatically generated by the Data Office taking into account the hepcidin results of the participants. The list will indicate the colour/code of the supplement the participant receives for the following 7 days but the field coordinator will not know which colour/code is allocated to which supplement. The sachets will be colour coded or numbered (2 codes for treatment arm A (12 mg and 12 mg iron), 2 codes for arm B (12 mg and 0 mg iron) and 2 codes for arm C (6 mg and 0 mg iron)) (Figure 2). The pre-packed weekly supplies labelled with each participant's ID will be handed over to the PI who is responsible for distribution to the field workers who will distribute to the individual participants and supervise consumption. The laboratory staff and data entry clerks will be blinded as well. See also Section 11.3 on participant confidentiality.

Blinding of participants as well as field staff is considered important as morbidity information will be collected which would be influenced by knowing if the supplement contains iron or not. Blinding of the PI, lab technicians and data entry clerks is important due to the weekly hepcidin screening.

The allocation of the colour code will be done by 2 people independent from the study and the key will be kept in a lockable cabinet in Keneba. The blinding for the study may be broken if in any of the 3 treatment arms, safety issues arise and we are advised by the ITM and DSMB to do so (Section 6).

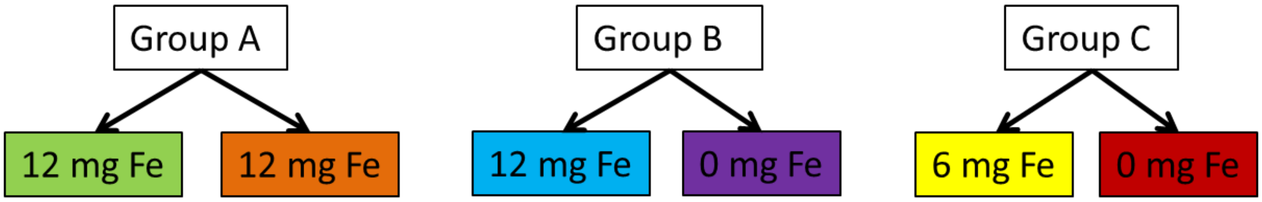

Figure 2: Example of blinding in case colour codes are used

3.3 Sub-studies

The sub-study investigating iron absorption using a stable isotope technique (see Appendix 2 for a Schematic of the sub-study design) will be conducted in 30 children in each study arm resulting in a total of 90 children, who will be enrolled in both the main study and this sub-study. The only difference to children only participating in the main study will be that these children will consume a dose of isotopically labelled iron at the time of recruitment and their participation in the main study will be delayed by at least 7 months. In addition, we will ask them for an additional venous blood sample 84 days after the main study has finished (Day 168).

Children, 6-8 months old, with informed consent for both the main and sub-study (separate participant information to be used for this group) obtained from their mothers/guardians and fulfilling all other criteria to participate in the main study (HAZ < -3, WAZ < -3, WHZ < -2, RDT negative for malaria) with the exception of Hb which needs

to be ≥ 7 and < 12 g/dL (main study criteria is Hb ≥7 and < 11 g/dL) assessed by HemoCue, will be given one oral dose of ^{57}Fe on the day of recruitment. The dose will consist of 2 mg iron in the form of ^{57}Fe-enriched ferrous sulphate and will be given 1h after a feeding and 1h before the next feeding. The iron will be dissolved in 10 mL of a 5% glucose solution and will be administered using a syringe directly into the mouth of the infant.

The children will start participating in the main study earliest 7 months after stable isotope administration. This period is needed to ensure that the administered stable isotope has homogeneously equilibrated with total body iron. We will take an additional venous blood sample 84 days after the end of the main study (Day 168) which will serve as a control for each subject (period without supplementation).

3.4 Investigational products or interventions

3 investigational products will be used in the study:

- MNP containing 12 mg iron
- MNP containing 6 mg iron
- MNP containing 0 mg iron

In the sub-study we will in addition administer a single dose of 2 mg iron in the form of ^{57}Fe-enriched ferrous sulphate dissolved in 10 mL of a 5% glucose solution.

3.4.1 Description of product or intervention

The nutritional supplement to be used in this trial is a MNP (MixMe WHO) produced by DSM and distributed by UNICEF and WFP. This product contains 10 mg of iron. For our study purposes the iron concentration will be altered and we will use 3 different products containing 12 mg, 6 mg or 0 mg iron/sachet (daily dose). Otherwise the composition is the same as in the MixMe.

In the sub-study, children will receive a single dose of 2 mg isotopically enriched elemental iron (^{57}Fe) dissolved in 10 mL of a 5% glucose solution.

3.4.2 Formulation, packaging and labelling

Each sachet will consist of the micronutrient powder as in Table 1 below. The different type of MNP will be produced by DSM South Africa under GMP conditions where it will also be dosed into the gusseted plastic/aluminum bags (Figure 3) bearing a label indicating Batch number, Material code, Pack size, Date of Manufacture, Best Before date, Addition rate and Storage conditions.

Table 1: Vitamins and minerals in a single daily dose (sachet)

Micronutrients	Dose/day
Vitamin A (ug RE)	400
Vitamin D (ug)	5
Vitamin E (mg)	5
Vitamin C (mg)	30
Thiamine (B_1) (mg)	0.5
Riboflavin (B_2) (mg)	0.5
Niacin (B_3) (mg)	6
Pyridoxine (B_6) (mg)	0.5

Cobalamine (B_{12}) (ug)	0.9
Folate (ug)	150
Iron (mg)	12 or 6 or 0
Zinc (mg)	4.1
Copper (mg)	0.56
Selenium (ug)	17
Iodine (ug)	90

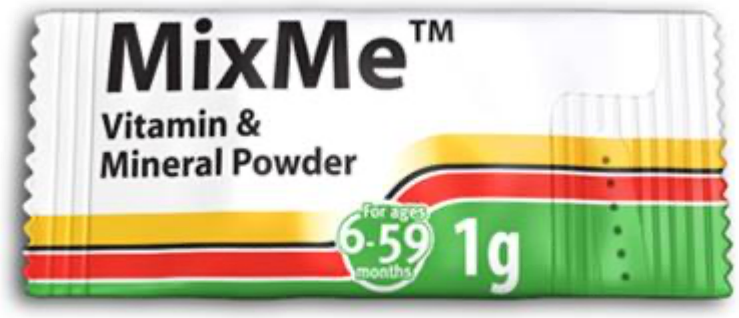

Figure 3: Single dose sachet as it will be used in the study. The sachets will be colour or number coded.

For the sub-study, the isotopically labeled $FeSO_4$ will be prepared from isotopically enriched elemental iron (^{57}Fe) according to a predefined Standard Operating Procedure according to good laboratory practice. Briefly, isotopically labeled $FeSO_4$ will be dissolved in diluted sulfuric acid of highest purity at ETH Zurich. The solution will be pre-dosed (exact weight recorded) for administration to individual subjects into 2.5 mL Teflon containers and shipped under cool conditions to the MRC Keneba. The doses will be stored in the refrigerator in Keneba at 5°C.

3.4.3 Product storage and stability

The products will be stored under controlled conditions (in an air-conditioned small storage house at around 20°C) at the MRC Keneba. The product is stable for 18 months if kept below 25°C.
The stable isotope doses are stable for a minimum of 6 months at 5°C.

3.5 Dosage, preparation and administration of investigational product or intervention

Each participant will receive 1 daily dose of the supplement which corresponds to 1 sachet per day. Each day field workers will be visiting each study participant to distribute and supervise administration. The mothers of the children will be advised to give it with a little bit of expressed breast milk, any other liquid or a spoon full of a slurry or porridge.

In the sub-study, on the day of administration, the pre-dosed stable isotope solution and the 5% glucose solution (produced in the laboratory of the MRC Keneba according to a predefined Standard Operating Procedure) will be taken to the field where the stable isotope dose and the 10 ml glucose solution will be mixed in a disposable plastic cup. The dose will be administered directly into the child's mouth using a syringe after a 3 h fast.

After dose administration the child is not allowed to consume anything for another hour. Spillage and regurgitation will be noted.

3.6 Concomitant medications/treatments

No specific medication is prohibited during the study.

4 Selection and withdrawal of participants

4.1 Selection of participants

393 healthy children, 6-24 mo old, will be identified through the immunization records at the health facilities of Jarra West and Kiang East (rural Gambia). After informed consent is obtained, a finger prick blood sample for Hb and RDT assessment will be taken. If they are eligible, they will be allocated to the study arms.
To children also participating in the sub-study (age inclusion criterion: 6-8 months old), the iron dose will be administered if they are eligible for the main study but they will only be finally randomized to the study arms > 7 months later when they start participating in the main study (Section 3.3).

4.2 Eligibility of participants

Participants must meet all of the inclusion criteria and none of the exclusion criteria to be eligible to participate in the trial.

4.2.1 Inclusion criteria

- Apparently healthy as judged by a study nurse at day of recruitment
- Age: 6 to 24 mo
- HAZ, WAZ and WHZ >-3 SD
- Hb ≥ 7 g/dL and < 11 g/dL (Hb ≥ 7 g/dL and < 12 g/dL for the sub-study)
- Free of malaria (RDT negative)
- Resident in the study area
- Ability and willingness to comply with the study protocol (weekly study visits with finger prick blood sampling)
- Informed consent given by parent or guardian

4.2.2 Exclusion criteria

- Congenital disorders
- Chronic disease
- Regular medication
- Currently participating in another study

4.3 Withdrawal of participants

A study participant will be discontinued from participation in the study if:

- Hb concentration falls < 7 g/dL
- Any clinical significant adverse event (AE), laboratory abnormality, intercurrent illness, or other medical condition or situation occurs such that continued participation in the study would not be in the best interest of the participant.
- Development of a chronic disease
- Participation in another study

For further details on participant's premature termination see corresponding section below.

Participants are free to withdraw from the study at any time without giving a reason.

5 Study procedures and evaluations

For an overview see Appendix 3 "Schedule of Events".

5.1 Study schedule

5.1.1 Study sensitisation

The Regional Health Team (RHT) of the Lower River Region (LRR) has already been engaged and the proposal discussed. The team has given its support to the study. When SCC and Ethics approval is secured, the PI together with the RHT and staff of the facilities responsible for the catchment areas of Soma and Kiang Kaiaf will be sensitised. A team comprising a member of the RHT, staff of the facilities lead by the PI will tour all the communities to sensitise them on the project. The community sensitisation will involve influential leaders and community health workers. The aim of these sensitisations and discussions is to garner support for the research project.

5.1.2 Screening and enrollment (Baseline)

Mothers/Guardians of young children identified using immunization records at the respective health centres will be invited to the respective health centres where the study will be explained. Once mothers/guardians of the child have signed the informed consent form, the child will be physically examined, height and weight will be measured and a finger prick blood sample for Hb and RDT testing will be taken if all other inclusion criteria and no exclusion criteria are fulfilled. If Hb is ≥7 and < 11 g/dL and the RDT is negative a venous blood sample (5 mL in total, divided into1.5 mL into EDTA, 1.5 mL into LH and 1.5 mL into ACD and 0.5 mL into serum blood collection tubes) will be collected. We will then also collect a stool sample from the child. Children with Hb < 7 g/dL will not be enrolled and referred to the regional health centre for treatment according to national guidelines. Children with Hb ≥ 11 will not be enrolled as they don't need iron. Malaria positive children (positive RDT and confirmation by blood film) will not be enrolled and treated according to national guidelines. Enrolled children will be randomized to one of the three study arms as described in section 3.2.1.

5.1.3 Follow-up

Trained field workers will be visiting all children daily during the 12 weeks supplementation period in order to supervise the MNP administration and to check on the children's health status. Twice weekly, morbidity data (including questions regarding fever, diarrhoea, vomiting, cough, any other illness, appetite and any mediation taken and assessment of body temperature) will be captured. If a child is found unwell, the study nurse will check on the child and decide on treatment/referral to the next health centre. Every week children will be screened using a finger prick blood sample (200 µL) to determine their hepcidin, Hb and malaria status. Hb and RDT testing will be directly conducted in the field whereas hepcidin analysis will be performed in the laboratory in Keneba. When hepcidin results are available on the next day the respective MNP for the next 7 days for each child will be provided to the field assistants. Children found with a positive RDT during the study will be further tested with a blood film and treated in case malaria is confirmed according to national guidelines. Any child with an Hb < 7 mg/dL will be excluded and referred to the next health centre for management according to national guidelines.

In detail (Table 2), each Field Assistant will be responsible for a cluster of communities comprising approximately 25 participants from the 100 children recruited at a time (Section 3.1). In each cluster the weekly finger prick blood samples will be collected in the morning of a predefined week day and sent to Keneba for hepcidin analysis. Hepcidin results will be available the following day at mid-day when the next 7 day's supplement supply for each child will be prepared by the field coordinator in Keneba according to a list generated by the Data Office (taking into account hepcidin results). The following morning the supply will be handed over to the FA to be distributed to the children for the next 7 days daily. Table 2 also shows the days of morbidity information collection.

Table 2: Daily study visits for each cluster and each batch of ≈ 100 participants (in addition to the daily supervised supplement administration visit). The visits will be the same for each week of the total of 12 weeks.

Cluster	Communities	Respon-sible FA	No. of children	Type of visit						
				Mon	*Tue*	*Wed*	*Thu*	*Fri*	*Sat*	*Sun*
1	Soma<br>Si Kunda	TBD	25	FP	MQ	SS		MQ		
2	Karantaba<br>Kani Kunda<br>Sankwia	TBD	25		FP	MQ	SS		MQ	
3	Mansakonko<br>Pakalinding<br>Jenoi	TBD	25			FP	MQ	SS		MQ
4	Kaiaf<br>Genieri<br>Jiffin<br>Toniataba	TBD	25	MQ			FP	MQ	SS	

TBD = to be determined
FP = finger prick sample collection
SS = new supplement supply (for next 7 days)
MQ = Morbidity questionnaire

On Days 14 and 84 another faecal sample and on Days 49 and 84 another venous blood sample (5 mL), instead of a finger prick blood sample, will be collected. All blood samples will be kept in a cool chest after collection and transported to Keneba on ice. An additional blood sample will be collected on Day 168 from the children also participating in the sub-study. Height and weight will be re-measured at Day 49, 84 and 168 (for the sub-study children).

Study evaluation and outcome measures are described in Section 8.2 (Statistical analysis).

5.1.4 Final study visit

The final study visit will be on Day 84 when the last venous blood sample and faecal sample is collected. For the children participating in the sub-study as well, the final study visit will be on Day 168 when the last venous blood sample will be collected. Follow-up of all AEs/SAEs will be continued until resolved (Section 6.1.4).

5.1.5 Early termination visit

An early termination visit may occur in this study because of a participant's voluntary withdrawal, trial team decision or at the discretion of the Data Safety Monitoring Board as described in Section 7. Apart from the safety evaluations, no other evaluations required for the final study visit will be done.

5.2 Study evaluations

The approach to the evaluation of this trial is a test of non-inferiority with the primary non-inferiority endpoint being Hb at day 84 (Section 8.2) to evaluate efficacy of arms B

and C when compared to arm A. Abnormal laboratory results indicative of severe anaemia and malaria positive will be reported as AE.

For further safety consideration see Section 6.

5.2.1 Clinical evaluations

Health status of the children at enrolment will be assessed through a physical examination by the study nurse. Height and weight will be measured at Days 0, 49 and 84 (and 168 for the children participating in the sub-study as well).

5.2.2 Laboratory evaluations

Blood samples: In the ***venous blood samples*** collected on Days 0, 49 and 84 full haematology (including reticulocytes, 400 µl from EDTA blood) (NB choice of a replacement haematology analyser for Keneba is to be decided), ZnPP (in washed RBCs from EDTA blood, 35 µl) using the Aviv Haematofluorometer, ferritin, sTfR, serum iron, TSAT, CRP, AGP (altogether, 500 µl LH plasma) using a fully automated biochemistry analyser (Cobas Integra 400 plus) and hepcidin (100 µl serum) using the Hepcidin-25 (human) EIA Kit (Bachem) and a Thermo Multiskan FC Microplate Photometer will be performed.

Ex vivo growth of *P. falciparum* will be assessed in washed RBCs (500 µl from ACD tube) using a field-ready 96-well plate method with FACS (basic 2-color FACS, BD Accuri) readout in which RBC size will also be assessed. A small subset of RBCs will be lysed for measurement of riboflavin status by the erythrocyte glutathione reductase activation coefficient (EGRAC) test because this may affect RBC stability.

Ex vivo growth of sentinel bacteria (*Staphylococcus aureus*, *Staphylococcus epidermidis* (from 300 µl LH plasma) and *Salmonella* Typhimurium (from 150 µl serum)) will be assessed by optical density plots confirmed by baseline and end-point colony counts using the cell culture counting facility of a Thermo-Scientific Nanodrop spectrophotometer.

DNA will be extracted from baseline EDTA whole blood samples (500 µl) using the salting out method according to the Keneba Biobank standardized procedures to look at genetics of iron metabolism. Known genetic risk factors to be assessed include the alpha-thalassemia, G6PD and sickle traits. Furthermore, putative functional and key tagging variants in iron regulatory and inflammatory pathways will be screened. Much of this will be available from the Illumina Infinium Human Exome Bead Chip (Exome Chip) that we are already using. A specific 'iron chip' may be developed.

All the above-mentioned analyses will be conducted in the laboratory in Keneba.

Genotyping for haemoglobinopathies in the Day 0 blood samples will be performed from EDTA blood (100 µl) at the MRC Fajara.

In the weekly ***finger prick blood samples*** Hb using a HemoCue 301 (5 µl blood) and an RDT (5 µl blood) will be directly performed in the field. In case of a positive RDT a blood film (20 µl) will be prepared and read at the laboratory in Keneba. Hepcidin from serum collected into a BD microtainer® will be measured in the Laboratory in Keneba as described above for venous blood.

Stool samples: Samples at Days 0, 14 and 84 will be collected. Stool samples will be collected in a sterile container with a tight, screw top lid that includes an Anerocult® sachet (Merck, Damrstadt, Germany) to create an anaerobic environment. The samples will be

labelled and frozen at -20°C. Calprotectin (Calprest, Eurospital, Trieste, Italy) and Zonulin (Immundiagnistik, Bernsheim, Germany) will be assessed using ELISA methods. The gut microbiota composition will be assessed with pyrosequencing and using qPCR on enterobacteriaceae and target commensal bacteria in a subset of 25 children in each study group at ETH Zurich.

Sub-study:
In addition to all the analysis done in the venous blood samples for the main study (Days 0, 49 and 84) isotopic ratio (500 µl RBC from LH blood) will be measured using an Inductively Coupled Plasma Mass Spectrometer (ICP-MS) at ETH Zurich. In the additional blood sample taken in this subgroup of participants on Day 168, full haematology, ZnPP, ferritin, sTfR, serum iron, TSAT, CRP and AGP will be measure as described above in Keneba and isotopic composition will be measured at ETH Zurich.

For possible future use of specimens see Section 11.4.

6 Safety considerations

This trial will be overseen by a Data Safety Monitoring Board (DSMB) (chaired by Dr Jay Berkley, KEMRI Wellcome Trust, Kilifi, Kenya). . The DSMB will be responsible for reviewing:

- the trial protocol (before the trial is started)
- all interim data from the trial
- treatment safety and efficacy including the protection of the rights and well-being of the participants
- the overall progress of the study

The DSMB will additionally review all Serious Adverse Events (SAE).

6.1 Methods and timing for assessing, recording, and analysing safety parameters

The trial will be conducted according to Good Clinical Practice (GCP) principles. The DSMB will determine how they will monitor the data and safety interest of the participants. The DSMB will also determine how and the frequency of its meetings.

6.1.1 Adverse events

An adverse event (AE) is defined as any untoward or unfavourable medical occurrence in a human subject, including signs and symptoms which are temporally associated with the subject's participation in the research, whether or not considered related to the subject's participation in the research. Participants will be monitored for AEs on each scheduled follow up day. All symptoms or signs reported or observed will be assessed by the study Field Assistant and will be recorded as an AE after evaluation by the study nurse. Persistently low Hb will be considered as an AE and will be followed up.

6.1.2 Serious adverse events (SAEs)

A **SAE** is any AE that is life-threatening or results in death or requires hospitalisation or prolongation of hospitalisation or is a persistent or significant disability/incapacity. All SAEs will be investigated by a physician.

6.1.3 Assessment of intensity

The PI, with support from the clinical team in Keneba, will assess the severity or intensity of the AEs and laboratory changes as follows and document it into the AE form:

Grade		Description
1	Mild	Awareness of sign or symptom, but easily tolerated
2	Moderate	Enough discomfort to cause interference with usual activity
3	Severe	Incapacitating with inability to work or do usual activity
4	Life-threatening	This grade will be considered as SAE

The term "severe" is often used to describe the intensity (severity) of a specific event (as in mild, moderate, or severe myocardial infarction); the event itself, however, may be of relatively minor medical significance (such as severe headache). This is not the same as "serious", which is based on the outcome or criteria defined under the SAE definition. An event can be considered serious without being severe if it conforms to the seriousness criteria; similarly severe events that do not conform to the criteria are not necessarily serious. Seriousness (not severity) serves as a guide for defining regulatory reporting obligations.

6.1.4 Assessment of causality

Every effort will be made by the PI and team to explain each AE and assess its causal relationship to administration of the investigational product. This explanation will be based on the type of event, the relationship of the event to the time of trial intervention, and the natural history of the underlying diseases, concomitant therapy, etc. The results will be documented on the AE form. The relationship of an AE to the investigational product will be assessed according to the following definitions:

Not related
- No temporal relationship to trial intervention; *and*
- Event could be explained by alternate aetiology (clinical state, environmental or other interventions).

Unlikely related
- Temporal relationship to trial intervention improbable (but not impossible); *but*
- Disease or other products provide plausible explanations.

Possibly Related
- Reasonable temporal relationship to trial intervention; *but*
- Event could also be explained by alternate aetiology (clinical state, environmental or other interventions);

Probably Related
- Reasonable temporal relationship to trial intervention; *and*
- Event could not be explained by alternate aetiology (clinical state, environment, or other interventions);

Definitely Related
- Reasonable temporal relationship to trial intervention; *and*
- Event could not be explained by alternate aetiology (clinical state, environmental or other interventions); *or*
- Event could be confirmed with a positive re-challenge test, where applicable.

The mothers/guardians of participating children will be instructed to contact the field assistant or a member of the study team, should the child manifest any signs or symptoms they perceive as severe during the period extending from performance of the first trial procedure to the end of the study.

All findings observed or reported from the day of the first administration of the investigational product will be recorded on an AE Form by the team. Whenever possible, AEs will be documented in terms of a diagnosis or syndrome rather than multiple symptoms that are clear manifestations of the same diagnosis/syndrome. In case signs and symptoms are reported by the participants, a medical diagnosis will be obtained by the PI. If a diagnosis cannot be obtained then each sign or symptom will be recorded as separate events.

The action taken (e.g. discontinuation of investigational product, withdrawal of the participant from the trial, requirement of concomitant medication or treatment, others) will be recorded on the AE Form. If hospitalization or its prolongation is required this will be reported as a SAE.

All AEs will be followed until resolution of the event and/or the end of the trial. The outcome will be assessed as follows:

- Resolved
- Resolved with sequelae
- Ongoing
- Death
- Lost to follow up

Treatment of any AE and SAE will be recorded in the appropriate section of the CRF.

6.2 Reporting procedures

The PI shall report all SAEs without filtration, whether or not related to the trial intervention, within 24 hours of becoming aware of the event to the DSMB and the Sponsor. If the SAE is related to the trial intervention, the Ethics Committee will be notified within 7 calendar days if fatal or life-threatening, and all others within 15 calendar days.

The minimum information required for this initial SAE report is:

- Trial number and (short) title
- Participant's ID
- Date and time of onset
- Description of the event (clinical history, associated signs and symptoms)
- Intervention administered
- Reporter's name

The PI will not wait for additional information to fully document the event before notifying. The report is then to be followed by submission of a completed SAE Report Form as soon as possible, detailing relevant aspects of the SAE in question. All actions taken by the PI and the outcome of the event must also be reported immediately.

For documentation of the SAE, any actions taken, outcome and follow-up, the SAE Report Forms will be used. All follow-up activities have to be reported, if necessary on one or more consecutive SAE report forms in a timely manner. All fields with additional or changed information must be completed and the report form should be forwarded to the DSMB within 5 calendar days after receipt of the new information. Hospital case records and autopsy reports, including verbal autopsy, will be obtained where applicable.

6.3 Safety oversight

Safety oversight shall be provided by a TM and a DSMB who will provide independent advice as stipulated in Section 6 above.

7 Discontinuation criteria

7.1 Participant's premature termination

Mothers/guardians of participating children have the right to stop their children's participation in the study at any time without giving a reason and this will not affect the medical care that would normally be received. The trial team may also withdraw a participant from the study if deemed necessary at any time taking in to consideration the reasons mentioned below. The reason for a participant's premature termination will be documented on the appropriate page of the CRF and specified which of the following possible reasons were responsible for the premature termination:

- Serious Adverse Event
- Adverse Event
- Participant's consent withdrawal
- Development of withdrawal criterion
- Protocol deviation
- Migrated/moved from the study area
- Lost to follow-up

A 'lost to follow-up' is any participant who completed all protocol specific procedures up to the administration of the investigational product or intervention, but was then lost during the study period to any further follow-up, with no safety information and no efficacy endpoint data ever became available.

In case the participant decides to withdraw participation or consent during the study, we will not work on participant's samples without permission, but any information already generated from the samples will be kept and used. The study clinician may also ask for tests for the participant's safety. The PI will inquire about the reason for any withdrawal and follow-up with the participant regarding any unresolved AEs.

For withdrawn participants no specific data will be collected. Subjects will not be replaced as our sample size calculation takes into account a dropout rate of 15%.

7.2 Study discontinuation

The rules for study termination will be set by the TM and DSMB during their first meeting.

8 Statistical considerations

8.1 Sample size determination

Based on a SD of 1.15 of the mean haemoglobin value of children at 24 wk of age from a trial conducted at Keneba, a sample size of 131 participants in each of the 3 arms is required using a 1-sided α of 2.5 and a Bonferroni correction to adjust for multiple testing. A total sample size of 393 children followed up for 12 weeks with a drop-out of less than 15% will provide 80% power to establish that:

1) arm B is non-inferior to arm A on the primary endpoint (Hb concentration at Day 84) defined as the upper 96.7% confidence limit for the difference in mean haemoglobin being not greater than 0.5 g/dL (non-inferiority margin), the smallest value considered to be of public health relevance

2) arm C is non-inferior to arm A at the same level as above

3) arm C is non-inferior to arm B at the same level as above

For the sub-study, the power calculation for iron absorption is based on the data from Fomon *et al*[14] in infants. It is estimated that approximately 20 subjects per group are required to resolve a difference in absorbed iron of 0.080 mg/d between the control and intervention period, based on a SD of 0.13 mg/d (paired t-test). The between group difference that we estimate being able to resolve is 0.12 mg/d absorbed Fe (unpaired t-test). Lower variability is expected in the calculated k_{abs} values, which are independent from haemoglobin, body weight, and iron status, the main contributors to variability in the calculation of iron absorption. Considering attrition, 30 subjects per group will be recruited.

8.2 Statistical analysis

Data will be analysed using SPSS software. The primary outcome data (Hb concentration at day 84) will be tested for non-inferiority performing per-protocol analyses. Further, we will test whether the withdrawals due to low Hb are balanced between the arms. If this is not the case we will include children withdrawn due to low Hb in the primary analysis as will be described in detail in the statistical analytical plan.

Secondary outcome analysis of continuous variables will be done by using one-way ANOVA, followed by unpaired t-tests taking into account Bonferroni correction to compare parameters at Days 0 and 84 between the 3 arms. Within arm comparison will be done by using paired t-tests comparing data between Day 0 and Day 84. Non-normally distributed data will be log transformed before testing. Differences in categorical variables between study arms will be tested using a X^2 test and the effect within study arms using the McNemar test. Significance will be set at $P<0.05$.
Morbidity data will be analysed using multiple regression analysis controlling for possible confounders.

Gut microbiota results will be analysed with ANOVA. Posthoc tests will be performed if the time treatment interaction is found to be significant.

Further exploratory analysis will be performed using ZnPP, TSAT, serum iron, CRP, AGP, calprotectin and zonulin.

In the sub-study, the linear regression of $\log {}^{57}A^t$ (${}^{57}A^t$ = abundance at time t) against time will be calculated for each subject belonging to the three different groups for the period of Day 0 to Day 84 (with a midpoint sample at day 49). The slope (k_{abs}) of the regression reflects the absorption of iron per unit of time. The difference in the slopes between the groups will be equivalent to the average iron absorption over time in each group (k_{abs} in groups A, B, and C, respectively).
The assumption of homogenous labelling will be tested by testing the linearity of the slope between Days 0, 49 and 84. In each subject, $k_{abs,i}$ will be calculated from the slope of the isotopic ratio decrease over the intervention period. $k_{abs,c}$ will also be calculated for the control period (Day 84 and 168). k_{abs} will be modelled using linear mixed models with time (intervention and control period) and treatment as fixed factors and subjects ID as random factors. The time treatment interaction on k_{abs} will be assessed. A similar but separate

analysis will be conducted on the total amount of iron absorbed, which will be estimated from the k_{abs} value in each subject, and the group and time effect will be modelled with linear mixed models. The models will be compared to the standard treatment of supplementation with 12 mg Fe/day.
Significance will be set at $P<0.05$.

9 Data handling and record keeping

9.1 Data management and processing

All protocol required data will be captured in Case Report forms (CRF) that will be completed for each included participant. The CRF will either be paper form or an electronic data capture or a combination of both (depending on the state of development of the electronic data capturing system in Keneba at the time the study starts). Data will be entered/checked by Data Entry Clerks with the supervision of the Data Manager.

On the CRF, a reference to the source document will be provided. Instructions for completing all forms, including the CRF, used in the study will be developed.

The following data will be recorded: Date of informed consent, personal data (ID, initials, date of birth), socioeconomic data, height and weight, information on health status and regarding participation in other studies, date and time of all venous and finger prick blood collections, date and time of all faecal sample collections, date and time of supplement administration, data on morbidity, lab results.

9.2 Source documents and access to source data

The PI will maintain appropriate medical and research records for this study in compliance with the principles of good clinical practice and regulatory and institutional requirements for the protection of confidentiality of participants. The study team members will have access to records.

The authorised representatives of the sponsor, the ethics committee(s) or regulatory bodies may inspect all documents and records required to be maintained by the investigator, including but not limited to, medical records (office, clinic, or hospital) for the participants in this study. The clinical study site will permit access to such records.

9.3 Protocol deviations

A protocol deviation (PD) is any noncompliance with the clinical trial protocol, principals of good clinical practice (GCP), or other protocol-specific requirements. The noncompliance may be either on the part of the participant or the investigator including the study team members, and may result in significant added risk to the study participant. As a result of a deviation, corrective actions will be developed and implemented promptly.

If a deviation from, or a change of, the protocol is implemented to eliminate an immediate hazard(s) to trial participant without prior ethics approval, the PI or designee will submit the implemented deviation or change, the reasons for it, and, if appropriate, the proposed protocol amendment(s) as soon as possible to the relevant independent ethics committee (IEC) for review and approval.

The PI or designee will document and explain any deviation from the approved protocol on the CRF, where appropriate, and record and explain any deviation in a file note or

deviation form that will be maintained as an essential document. Deviations from the protocol, principals of GCP or trial specific requirements that might have an impact on the conduct of the trial or the safety of participants will be reported to the sponsor immediately and within 5 working days to the relevant IEC, the independent trial monitor, the DSMB as applicable.

10 Quality control and quality assurance

Quality control will be applied to each stage of the study. It will be the responsibility of the PI or designated trial team member to ensure that all source documents and CRFs are reviewed for accuracy and completeness. Any correction will be accurately accounted for.

Finger prick and venous blood collection will be done by the trial nurse. Collection of all samples will be recorded on the CRF.

All Field assistants and their supervisors including trial nurses will be trained. Selected participants from the RCH teams will also be trained. Further refresher training will be conducted as the trial progresses. Weekly meetings of the entire trial team will be convened in order to discuss al problems and lessons from the trial.

10.1 Study monitoring

Site monitoring will be conducted by a trained and experienced nurse of MRC-ING, Kenaba who is independent of the study.

11 Ethical considerations

This study will be conducted in accordance with the principles of good clinical practice (GCP).

11.1 General considerations on human subject protection

The study population is young children to who's mothers/guardians the study protocol will be explained orally in the presence of a witness in case they are illiterate or in writing and we will not start any study specific procedure before informed consent is obtained. The study investigates a nutritional supplement which has been specifically developed for young children of the age 6-59 months and used in many research projects. Participants will give informed consent to genetics testing of iron-related genes. Participants will not get any remuneration but will have free basic medical care for the duration of the study.

11.1.1Rationale for participant selection

Although anaemia affects all population groups amongst those at highest risk are young children. The anaemia prevalence rate is > 50% and remains a major public health problem in young children in the Gambia. The consequences of anaemia and iron deficiency include impaired neurocognitive development and immunocompetence leading to substantial loss of human potential.

Combating anaemia due to iron deficiency is a challenge due to the potential negative side-effects of iron when given to people with infections, as is extremely common in young children in The Gambia. Thus, young children are the most affected population of both iron deficiency and infection and would benefit most from a screen-and-treat approach. This approach would minimise exposure to iron in too high quantities and at the wrong time which would maximise the absorption and utilisation of iron if given at the right time.

11.1.2 Evaluation of risks and benefits

There are risks associated with a large intake of iron supplements especially in areas of malaria endemicity. The dose of iron given daily in the reference arm (12 mg) is according to WHO guidelines for 6-24 mo old children in non-malarious areas or malaria-endemic areas where it should be implemented in conjunction with measures to prevent, diagnose and treat malaria. This is the maximum of iron administered in this trial. As a recent study has shown that even at this dose adverse events may occur, we will closely monitor our study children. Field assistants will visit each child daily in order to supervise supplement administration but also to check on the child's health. In case of a fever a malaria rapid test will be performed and if positive the child will be treated according to national guidelines. A sick child will always be visited by a nurse for further clinical investigations and if needed referred to the nearest Health Centre. The group receiving only 6 mg iron daily and only if their hepcidin value is below the cut-off might be at risk of receiving too little iron. Weekly monitoring of Hb and malaria parasitaemia will ensure that children with an Hb below 7 g/dL will be excluded and treated according to national guidelines. Participants will experience some transient pain during blood sampling, which will be minimized by recruiting well-trained nurses for the study.
Children will benefit from daily monitoring of their health and from immediate care in case of illness. Although the mechanism of combating iron deficiency is problematic, it is clear that iron is a key micronutrient for the development of the immune system and cognitive function in these young children. Micronutrient powders as used in this study have shown to reduce anaemia and iron deficiency significantly in several studies. In addition, children in the study will benefit from the supplementation of not only iron but other key micronutrients related to healthy growth and development.

11.2 Informed consent

All field workers taking part in the recruitment of participants will be trained on translating the contents of the information sheet and the details of it will be explained to illiterate mothers/guardians in a language they understand in the presence of an independent literate witness. The literate mothers/guardians will be allowed to read the information sheet in their own time. They will be given enough time to ask questions and decide if they want their child to participate. Informed consent will be recorded by a signature or thumbprint on the consent form.

11.3 Participant confidentiality

To each participating child an individual identification (ID) number will be allocated. The participant ID numbers will be used on all samples, forms and on the CRF during the course of the study. Following sample collection and data entry, linkage of the ID back to the study participant will not be possible without a lookup table, which will only be held by the data manager and the designated data staff during the course of the study. Once data collection is complete, analysis will be performed on an anonymised copy of the data. At all stages, staff/collaborators responsible for sample analysis will be blinded as to the subject's identification. Together, these processes will ensure complete confidentiality of the data gathered and impartiality of data analysis.

11.4 Future use of stored specimen

Some of the blood and stool samples will be transferred to ETH Zurich, Switzerland for analysis because we don't have the equipment required for measuring all of the factors we are investigating in the Gambia.

DNA samples will be kept for future use to look at iron-related genes. Subjects will give consent to this within the informed consent used for the study. Remaining serum sample aliquots (if any) will be kept for future use in case this study results in another related research question. The DNA samples will be transferred to ING in London. The remaining serum samples will be kept in Keneba.

12 Financing and insurance

The research related costs of the proposed trial will be paid by MRC ING and the Bill & Melinda Gates Foundation. It is not a regulatory requirement in The Gambia that participants be insured for such a trial.

13 Publication policy

At least two publications in peer-reviewed journals will be written. Findings will also be presented at international conferences and shared with the National Nutrition Agency [NaNA] and the Ministry of Health of The Gambia.

14 References

1. Lozoff B, Smith JB, Kaciroti N, Clark, KM, Guevara S, Jimanez E. Functional significance of early-life iron deficiency: Outcomes at 25 years. J Pediatr 2013;163:1260-6.
2. Sazawal S, Black RE, Ramsan M Chwaya HM, Stoltzfus RJ, Dutta A, Dhingra U, Kabole I, Deb S, Othman MK, et al. Effects of routine prophylactic supplementation with iron and folic acid on admission to hospital and mortality in preschool children in a high malaria transmission setting: community-based, randomised, placebo-controlled trial. Lancet 2006;367:133-43.
3. WHO Secretariat on behalf of the participants to the consultation. Conclusions and recommendations of the WHO Consultation on prevention and control of iron deficiency in infants and young children in malaria-endemic areas. Food Nutr Bull 2007;28:S621-7.
4. Soofi S, Cousens S, Iqbal SP, Akhund T, Khan J, Ahmed I, Zaidi AK, Bhutta ZA. Effect of provision of daily zinc and iron with several micronutrients on growth and morbidity among young children in Pakistan: a cluster-randomised trial. Lancet 2013;382:29-40.
5. Prentice AM, Ghattas H, Cox SE. Host-pathogen interactions: can micronutrients tip the balance? J Nutr. 2007;137:1334-7
6. Veenemans J, Milligan P, Prentice AM, Schouten LRA, Inja N, van der Heijden AC, de Boer LCC, Jansen EJS, Koopmans AE, Enthoven WTM, et al. Effect of supplementation with zinc and other micronutrients on malaria in Tanzanian children: a randomised trial. PLoSMed 2011;11:e1001125.
7. Zimmermann MB, Chassard C, Rohner F, N'goran EK, Nindjin C, Dostal A, Utzinger J, Ghattas H, Lacroix C, Hurrell RF. The effects of iron fortification on the gut microbiota in African children: a randomized controlled trial in Cote d'Ivoire. Am J Clin Nutr. 2010;92:1406-15.

8. Zlotkin S, Newton S, Aimone AM, Azindow I, Amenga-Etego S, Tchum K, Mahama E, Thorpe KE, Owusu-Agyei S. Effect of iron fortification on malaria incidence in infants and young children in Ghana: a randomized trial. JAMA 2013;310:938-47.
9. Ganz T. Hepcidin and iron regulation, 10 years later. Blood. 2011;117:4425-4433.
10. Prentice AM, Doherty CP, Abrams SA, et al. Hepcidin is the major predictor of erythrocyte iron incorporation in anemic African children. Blood. 2012;119:1922-1928.
11. Drakesmith H, Prentice A. Hepcidin and the iron/infection axis. Science 2012;338:768-772.
12. De-Regil LM, Suchdev PS, Vist GE, Walleser S, Pena-Rosas JB. Home fortification of foods with multiple micronutrient powders for health and nutrition in children under two years of age. Cochrane Database Syst Rev. 2011;(9):CD008959.
13. WHO. Guidline: Use of multiple micronutrient powders for home fortification of foods consumed by infants and children 6-23 months of age. 2011. World Health Organization, Geneva.
14. Fomon SJ, Nelson SE, Serfass RE, Ziegler EE. Absorption and loss of iron in toddlers are highy correlated. J Nutr. 2005;135:771-7.

Appendix 1: Schematic of study design - main study

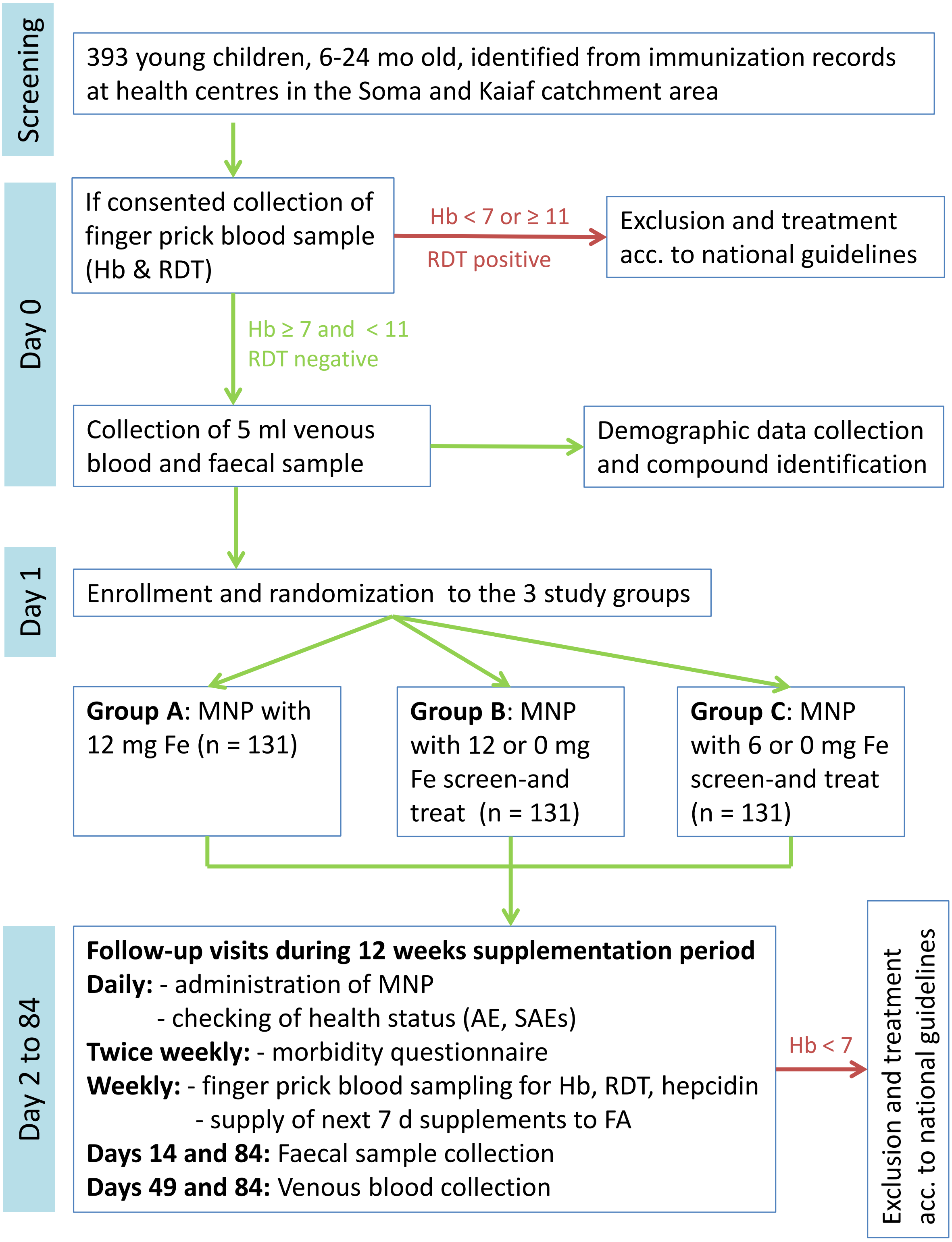

Appendix 2: Schematic of study design – sub-study

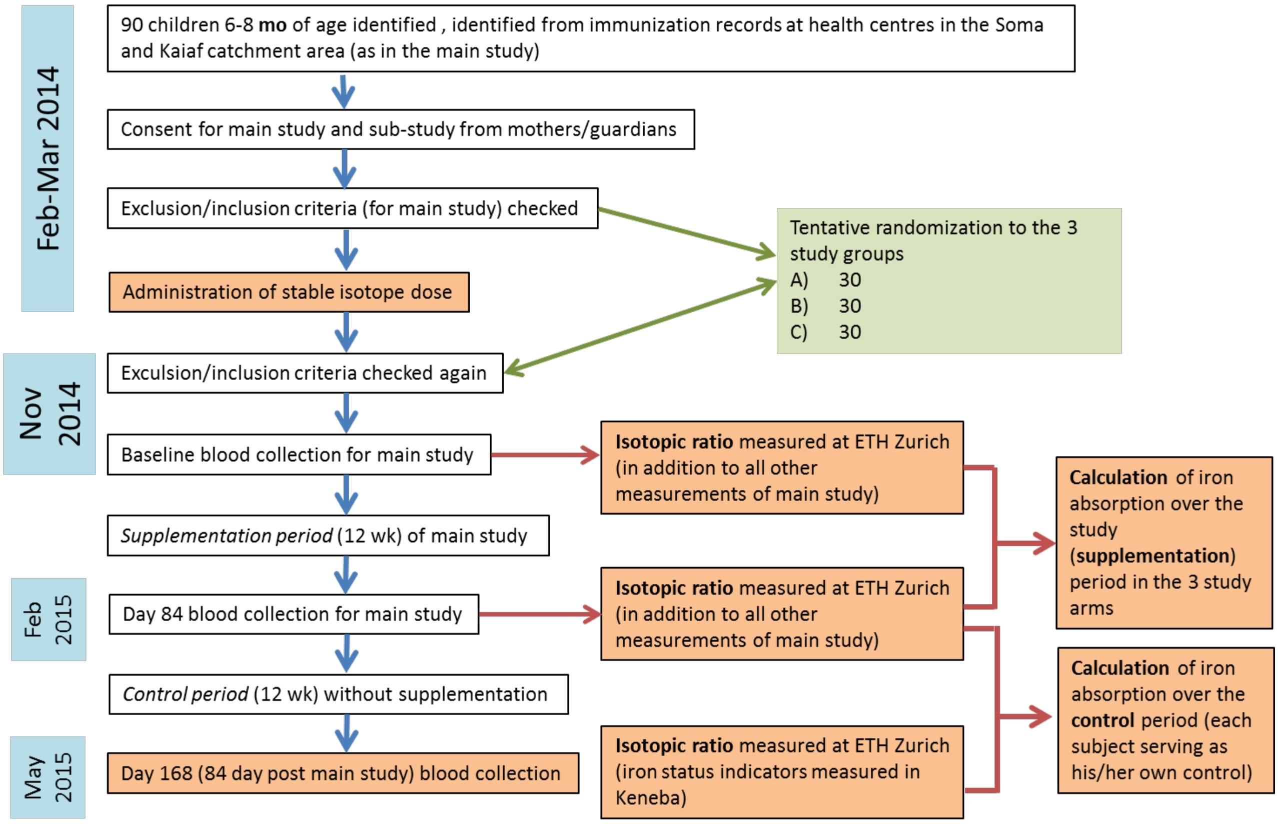

Appendix 3: Schedule of events

				Follow-up schedule													
Procedures		Screening	Baseline (Day 0)	Study wk 1 (Day	2 (Day 14)	3 (Day 21)	4 (Day 28)	5 (Day 35)	6 (Day 42)	7 (Day 49)	8 (Day 56)	9 (Day 63)	10 (Day 70)	11 (Day 77)	12 (Day 84)	Day 168 (Sub-study)	Discontinuation
Identification of participants		×															
Consent/Enrolment		×	×														
Venous blood (5mL)			×														
Faecal sample			×														
Randomization			×														
Demographic data		×															
Study intervention	Finger prick			×	×	×	×	×	×		×	×	×	×			
	Venous blood		×							×					×	×	
	Faecal sample				×										×		
	Supplements		7×	7×	7×	7×	7×	7×	7×	7×	7×	7×	7×	7×	7×		
	Morbidity questionnaire			2×	2×	2×	2×	2×	2×	2×	2×	2×	2×	2×	2×		
Assessment of Adverse Events	Hb levels		×	×	×	×	×	×	×	×	×	×	×	×	×		×
	Malaria		×	×	×	×	×	×	×	×	×	×	×	×	×		×
	Hepcidin		×	×	×	×	×	×	×	×	×	×	×	×	×		×
	SAEs			×	×	×	×	×	×	×	×	×	×	×	×		×

Appendix 4: Declaration of Helsinki

WORLD MEDICAL ASSOCIATION DECLARATION OF HELSINKI
Ethical Principles for Medical Research Involving Human Subjects

Adopted by the 18th WMA General Assembly, Helsinki, Finland, June 1964, and amended by the:
29th WMA General Assembly, Tokyo, Japan, October 1975
35th WMA General Assembly, Venice, Italy, October 1983
41st WMA General Assembly, Hong Kong, September 1989
48th WMA General Assembly, SomersetWest, Republic of South Africa, October 1996
52nd WMA General Assembly, Edinburgh, Scotland, October 2000
53rd WMA General Assembly,Washington, DC, USA, October 2002 (Note of Clarification added)
55th WMA General Assembly, Tokyo, Japan, October 2004 (Note of Clarification added)
59th WMA General Assembly, Seoul, Republic of Korea, October 2008
64th WMA General Assembly, Fortaleza, Brazil, October 2013

Preamble

1. The World Medical Association (WMA) has developed the Declaration of Helsinki as a statement of ethical principles for medical research involving human subjects, including research on identifiable human material and data.
   The Declaration is intended to be read as a whole and each of its constituent paragraphs should be applied with consideration of all other relevant paragraphs.
2. Consistent with the mandate of the WMA, the Declaration is addressed primarily to physicians. The WMA encourages others who are involved in medical research involving human subjects to adopt these principles.

General Principles

3. The Declaration of Geneva of the WMA binds the physician with the words, "The health of my patient will be my first consideration," and the International Code of Medical Ethics declares that, "A physician shall act in the patient's best interest when providing medical care."
4. It is the duty of the physician to promote and safeguard the health, well-being and rights of patients, including those who are involved in medical research. The physician's knowledge and conscience are dedicated to the fulfilment of this duty.
5. Medical progress is based on research that ultimately must include studies involving human subjects.
6. The primary purpose of medical research involving human subjects is to understand the causes, development and effects of diseases and improve preventive, diagnostic and therapeutic interventions (methods, procedures and treatments). Even the best proven interventions must be evaluated continually through research for their safety, effectiveness, efficiency, accessibility and quality.
7. Medical research is subject to ethical standards that promote and ensure respect for all human subjects and protect their health and rights.
8. While the primary purpose of medical research is to generate new knowledge, this goal can never take precedence over the rights and interests of individual research subjects.
9. It is the duty of physicians who are involved in medical research to protect the life, health, dignity, integrity, right to self-determination, privacy, and confidentiality of personal information of research subjects. The responsibility for the protection of research subjects must always rest with the physician or other health care professionals and never with the research subjects, even though they have given consent.
10. Physicians must consider the ethical, legal and regulatory norms and standards for research involving human subjects in their own countries as well as applicable international norms and standards. No national or international ethical, legal or

regulatory requirement should reduce or eliminate any of the protections for research subjects set forth in this Declaration.

11. Medical research should be conducted in a manner that minimises possible harm to the environment.
12. Medical research involving human subjects must be conducted only by individuals with the appropriate ethics and scientific education, training and qualifications. Research on patients or healthy volunteers requires the supervision of a competent and appropriately qualified physician or other health care professional.
13. Groups that are underrepresented in medical research should be provided appropriate access to participation in research.
14. Physicians who combine medical research with medical care should involve their patients in research only to the extent that this is justified by its potential preventive, diagnostic or therapeutic value and if the physician has good reason to believe that participation in the research study will not adversely affect the health of the patients who serve as research subjects.
15. Appropriate compensation and treatment for subjects who are harmed as a result of participating in research must be ensured.

Risks, Burdens and Benefits

16. In medical practice and in medical research, most interventions involve risks and burdens.

    Medical research involving human subjects may only be conducted if the importance of the objective outweighs the risks and burdens to the research subjects.
17. All medical research involving human subjects must be preceded by careful assessment of predictable risks and burdens to the individuals and groups involved in the research in comparison with foreseeable benefits to them and to other individuals or groups affected by the condition under investigation.

    Measures to minimise the risks must be implemented. The risks must be continuously monitored, assessed and documented by the researcher.
18. Physicians may not be involved in a research study involving human subjects unless they are confident that the risks have been adequately assessed and can be satisfactorily managed.

    When the risks are found to outweigh the potential benefits or when there is conclusive proof of definitive outcomes, physicians must assess whether to continue, modify or immediately stop the study.

Vulnerable Groups and Individuals

19. Some groups and individuals are particularly vulnerable and may have an increased likelihood of being wronged or of incurring additional harm.

    All vulnerable groups and individuals should receive specifically considered protection.
20. Medical research with a vulnerable group is only justified if the research is responsive to the health needs or priorities of this group and the research cannot be carried out in a non-vulnerable group. In addition, this group should stand to benefit from the knowledge, practices or interventions that result from the research.

Scientific Requirements and Research Protocols

21. Medical research involving human subjects must conform to generally accepted scientific principles, be based on a thorough knowledge of the scientific literature, other relevant sources of information, and adequate laboratory and, as appropriate, animal experimentation. The welfare of animals used for research must be respected.
22. The design and performance of each research study involving human subjects must be clearly described and justified in a research protocol. The protocol should contain a statement of the ethical considerations involved and should indicate how the principles in this Declaration have been addressed.

    The protocol should include information regarding funding, sponsors, institutional affiliations, potential conflicts of interest, incentives for subjects and information

regarding provisions for treating and/or compensating subjects who are harmed as a consequence of participation in the research study.
In clinical trials, the protocol must also describe appropriate arrangements for post-trial provisions.

Research Ethics Committees

23. The research protocol must be submitted for consideration, comment, guidance and approval to the concerned research ethics committee before the study begins. This committee must be transparent in its functioning, must be independent of the researcher, the sponsor and any other undue influence and must be duly qualified. It must take into consideration the laws and regulations of the country or countries in which the research is to be performed as well as applicable international norms and standards but these must not be allowed to reduce or eliminate any of the protections for research subjects set forth in this Declaration.
The committee must have the right to monitor ongoing studies. The researcher must provide monitoring information to the committee, especially information about any serious adverse events. No amendment to the protocol may be made without consideration and approval by the committee. After the end of the study, the researchers must submit a final report to the committee containing a summary of the study's findings and conclusions.

Privacy and Confidentiality

24. Every precaution must be taken to protect the privacy of research subjects and the confidentiality of their personal information.

Informed Consent

25. Participation by individuals capable of giving informed consent as subjects in medical research must be voluntary. Although it may be appropriate to consult family members or community leaders, no individual capable of giving informed consent may be enrolled in a research study unless he or she freely agrees.
26. In medical research involving human subjects capable of giving informed consent, each potential subject must be adequately informed of the aims, methods, sources of funding, any possible conflicts of interest, institutional affiliations of the researcher, the anticipated benefits and potential risks of the study and the discomfort it may entail, post-study provisions and any other relevant aspects of the study. The potential subject must be informed of the right to refuse to participate in the study or to withdraw consent to participate at any time without reprisal. Special attention should be given to the specific information needs of individual potential subjects as well as to the methods used to deliver the information.
After ensuring that the potential subject has understood the information, the physician or another appropriately qualified individual must then seek the potential subject's freely-given informed consent, preferably in writing. If the consent cannot be expressed in writing, the non-written consent must be formally documented and witnessed.
All medical research subjects should be given the option of being informed about the general outcome and results of the study.
27. When seeking informed consent for participation in a research study the physician must be particularly cautious if the potential subject is in a dependent relationship with the physician or may consent under duress. In such situations the informed consent must be sought by an appropriately qualified individual who is completely independent of this relationship.
28. For a potential research subject who is incapable of giving informed consent, the physician must seek informed consent from the legally authorised representative. These individuals must not be included in a research study that has no likelihood of benefit for them unless it is intended to promote the health of the group represented by the potential subject, the research cannot instead be performed with persons

capable of providing informed consent, and the research entails only minimal risk and minimal burden.

29. When a potential research subject who is deemed incapable of giving informed consent is able to give assent to decisions about participation in research, the physician must seek that assent in addition to the consent of the legally authorised representative. The potential subject's dissent should be respected.
30. Research involving subjects who are physically or mentally incapable of giving consent, for example, unconscious patients, may be done only if the physical or mental condition that prevents giving informed consent is a necessary characteristic of the research group. In such circumstances the physician must seek informed consent from the legally authorised representative. If no such representative is available and if the research cannot be delayed, the study may proceed without informed consent provided that the specific reasons for involving subjects with a condition that renders them unable to give informed consent have been stated in the research protocol and the study has been approved by a research ethics committee. Consent to remain in the research must be obtained as soon as possible from the subject or a legally authorised representative.
31. The physician must fully inform the patient which aspects of their care are related to the research. The refusal of a patient to participate in a study or the patient's decision to withdraw from the study must never adversely affect the patient-physician relationship.
32. For medical research using identifiable human material or data, such as research on material or data contained in biobanks or similar repositories, physicians must seek informed consent for its collection, storage and/or reuse. There may be exceptional situations where consent would be impossible or impracticable to obtain for such research. In such situations the research may be done only after consideration and approval of a research ethics committee.

Use of Placebo

33. The benefits, risks, burdens and effectiveness of a new intervention must be tested against those of the best proven intervention(s), except in the following circumstances:

    Where no proven intervention exists, the use of placebo, or no intervention, is acceptable; or

    Where for compelling and scientifically sound methodological reasons the use of any intervention less effective than the best proven one, the use of placebo, or no intervention is necessary to determine the efficacy or safety of an intervention and the patients who receive any intervention less effective than the best proven one, placebo, or no intervention will not be subject to additional risks of serious or irreversible harm as a result of not receiving the best proven intervention.

    Extreme care must be taken to avoid abuse of this option.

Post-Trial Provisions

34. In advance of a clinical trial, sponsors, researchers and host country governments should make provisions for post-trial access for all participants who still need an intervention identified as beneficial in the trial. This information must also be disclosed to participants during the informed consent process.

Research Registration and Publication and Dissemination of Results

35. Every research study involving human subjects must be registered in a publicly accessible database before recruitment of the first subject.
36. Researchers, authors, sponsors, editors and publishers all have ethical obligations with regard to the publication and dissemination of the results of research. Researchers have a duty to make publicly available the results of their research on human subjects and are accountable for the completeness and accuracy of their reports. All parties should adhere to accepted guidelines for ethical reporting. Negative and inconclusive as well as positive results must be published or otherwise made publicly available. Sources of funding, institutional affiliations and conflicts of interest must be declared

in the publication. Reports of research not in accordance with the principles of this Declaration should not be accepted for publication.

Unproven Interventions in Clinical Practice

37. In the treatment of an individual patient, where proven interventions do not exist or other known interventions have been ineffective, the physician, after seeking expert advice, with informed consent from the patient or a legally authorised representative, may use an unproven intervention if in the physician's judgement it offers hope of saving life, re-establishing health or alleviating suffering. This intervention should subsequently be made the object of research, designed to evaluate its safety and efficacy. In all cases, new information must be recorded and, where appropriate, made publicly available.
